# Disulfide-Bond-Induced Structural Frustration and Dynamic Disorder in a Peroxiredoxin from MAS NMR

**DOI:** 10.1021/jacs.3c01200

**Published:** 2023-05-04

**Authors:** Laura Troussicot, Alicia Vallet, Mikael Molin, Björn M. Burmann, Paul Schanda

**Affiliations:** † Department of Chemistry and Molecular Biology, University of Gothenburg, SE-405 30 Göteborg, Sweden; ‡ Wallenberg Centre for Molecular and Translational Medicine, University of Gothenburg, SE-405 30 Göteborg, Sweden; § Department of Life Sciences, Chalmers University of Technology, SE-405 30 Göteborg, Sweden; ∥ Institut de Biologie Structurale, Univ. Grenoble Alpes, CEA, CNRS, IBS, 71 Avenue des Martyrs, F-38044 Grenoble, France; ⊥ Institute of Science and Technology Austria, Am Campus 1, A-3400 Klosterneuburg, Austria

## Abstract

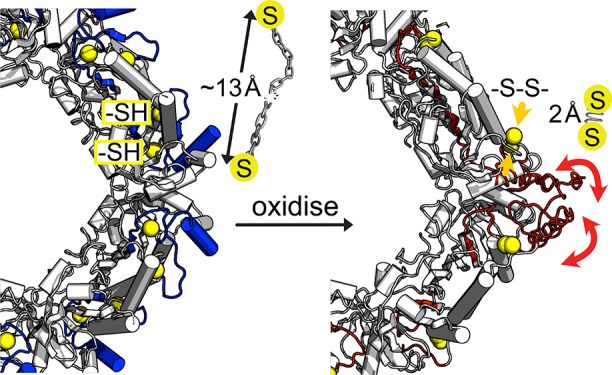

Disulfide bond formation is fundamentally important for protein structure and constitutes a key mechanism by which cells regulate the intracellular oxidation state. Peroxiredoxins (PRDXs) eliminate reactive oxygen species such as hydrogen peroxide through a catalytic cycle of Cys oxidation and reduction. Additionally, upon Cys oxidation PRDXs undergo extensive conformational rearrangements that may underlie their presently structurally poorly defined functions as molecular chaperones. Rearrangements include high molecular-weight oligomerization, the dynamics of which are, however, poorly understood, as is the impact of disulfide bond formation on these properties. Here we show that formation of disulfide bonds along the catalytic cycle induces extensive μs time scale dynamics, as monitored by magic-angle spinning NMR of the 216 kDa-large Tsa1 decameric assembly and solution-NMR of a designed dimeric mutant. We ascribe the conformational dynamics to structural frustration, resulting from conflicts between the disulfide-constrained reduction of mobility and the desire to fulfill other favorable contacts.

## Introduction

Peroxiredoxin enzymes (PRDXs), present in all kingdoms of life, play a key role in detoxifying cells from a variety of peroxides^1−4^ and act as peroxide sensors and protein redox regulation factors.^5^ Tsa1 is a typical 2-Cys peroxiredoxin from yeast, with two conserved cysteines that participate in the catalytic cycle: the peroxidatic cysteine (C_P_, Cys47) is responsible for the catalytic activity by reacting with a peroxide, ROOH, to produce the reduced, nontoxic form, ROH, thereby getting itself oxidized to a sulfenic acid, C_P_–SOH. To regenerate the peroxidatic cysteine, the so-called resolving cysteine (C_R_, Cys170 in Tsa1) forms a disulfide bond with the oxidized C_P_, thereby releasing a water molecule. The disulfide, C_P_–S–S–C_R_, is reduced to the sulfhydryl form, C_P_–SH and C_R_–SH, by the NADPH-dependent Trx/TrxR system.^4^ The catalytic cycle is schematically depicted in Figure S1. During this cycle, the protein undergoes a number of structural modifications, which have been investigated by crystallography. Structures of oxidized and reduced forms, enabled in some cases by mutation of the peroxidatic cysteine, have been reported for several PRDXs (reviewed in refs (4 and 6)). However, in crystal structures of oxidized states, large parts (>20 residues) have not been modeled, in particular around the cysteines, which has hampered the assessment of the structural and dynamical consequences of disulfide bond formation.^7,8^ For Tsa1 only the structure of a mutant has been reported, in which the catalytic cysteine C_P_ is mutated to a serine (C47S), and which therefore cannot undergo the functional cycle. PRDXs switch between different oligomeric states (dimers, decamers and higher oligomerization states) depending on redox state, pH, and other factors. The dimer–decamer transition has even been observed directly in cells.^9^ The dimer–decamer equilibrium is a complex and not entirely understood function of the above-mentioned parameters. Most of the available crystal structures report decameric rings, formed by the assembly of five dimers.

Exciting discoveries over the past years have brought to light an additional role of PRDXs: they can act as chaperones, preventing other proteins from aggregation. Jang et al. demonstrated that cytosolic Tsa1/Tsa2 can reversibly assemble to high-molecular weight (HMW) species *in vivo*, and that the HMW species of Tsa1 inhibit aggregation of citrate synthase three times more efficiently than the small heat-shock protein alphaB-Crystallin.^10^ The conversion to high-molecular weight species of cytosolic Tsa1/Tsa2 *in vivo* is favored by exposing the cell to H_2_O_2_ and heat shock, and (at least in the former case) is thought to be due to oxidation of the catalytic cysteine thiol into a sulfinic acid, Cys–SO(OH).^10−12^ Further studies of the Tsa1 chaperone function suggest a requirement for both HMW assembly and dissociation, the latter accompanied by Tsa1 desulfinylation, for efficient protein aggregate resolution *in vivo*,^13,14^ pointing to a more complex, dynamic regulation of both oligomeric and cysteine oxidation states underlying PRDX chaperone activity. Additional factors, including phosphorylation (Thr90 in hPrxI)^15^ or exposure to low pH,^16^ have been shown to induce a functional switch of PRDXs from peroxidases to chaperones. Since these seminal discoveries of a chaperone function, a similar behavior has been found in other 2-Cys PRDXs in different organisms such as SmPrx1 from the parasite *Schistosoma mansoni*,^17,18^ the human hPrx1,^15^ and the mitochondrial PRDX from *Leishmania infantum* mTXNPx.^19−21^ The ATP-independent chaperone activity was generally found *in vitro* by biochemical assays; for example, mTXNPx prevents aggregation of citrate synthase at elevated temperatures.^20^ Understanding the structural and dynamical consequences of disulfide formation and of oligomerization appears necessary to understand peroxidase function as well as the intriguing chaperone activity of PRDXs.

In this study we use magic-angle spinning (MAS) nuclear magnetic resonance (NMR) spectroscopy to probe dynamics of decameric Tsa1 in its reduced and oxidized forms. Recently developed NEar-rotary Resonance Relaxation Dispersion (NERRD) experiments demonstrate that disulfide formation induces extensive microsecond (μs) motions. Moreover, Bloch–McConnell *R*_1ρ_ relaxation dispersion MAS NMR reveals μs dynamics in the dimer–dimer interface. A mutation in this interface converts the protein to its dimeric form. Solution-NMR of this dimeric variant shows disorder in the vicinity of the cysteines, suggesting that the dynamic patch observed in oxidized decameric Tsa1 is at least in part present already in the dimer. We find a striking coincidence of the μs dynamics and structural frustration^22^ and propose that the induced dynamics in the oxidized form is important for the functional catalytic cycle, and possibly for chaperone function.

## Results and Discussion

### MAS NMR reveals that decameric reduced Tsa1 is overall rigid

We have performed size-exclusion chromatography coupled to multiangle light scattering (SEC-MALS) and found that at ambient temperature Tsa1 exists primarily in a decameric state, accounting for more than 80% of the total protein concentration (216 kDa; Supplementary Figure S2A). When reinjecting the decameric species into SEC-MALS, only the decameric state is obtained (Figure S2B), which establishes that we can obtain stable homogeneous decamer samples. We have reinjected the decamer also in the presence of a reducing agent (tris(2-carboxyethyl)phosphine; TCEP), as well as after treating the sample with H_2_O_2_, and found that under all these conditions Tsa1 forms a decameric assembly of ca. 216 kDa (Figure S2). Based on the slightly altered elution profile upon TCEP-addition leading to two distinct peaks representing the oxidized and reduced decameric species (Figure S2B), we conclude that the recombinant expressed Tsa1 is in the oxidized state.

NMR is ideally suited to probe dynamics and local conformation at the level of individual atoms, but in solution the NMR signals of particles as large as decameric Tsa1 are broadened due to the associated slow overall tumbling. Indeed, a solution-NMR ^1^H–^15^N spectrum of Tsa1 at ambient temperature shows only very few peaks, even with a deuterated sample and TROSY experiments^23^ (Figure S3). The ^1^H–^15^N peaks observed at ambient temperature correspond to the flexible loop regions, according to the chemical-shift assignment (discussed below).

**Figure 1:**
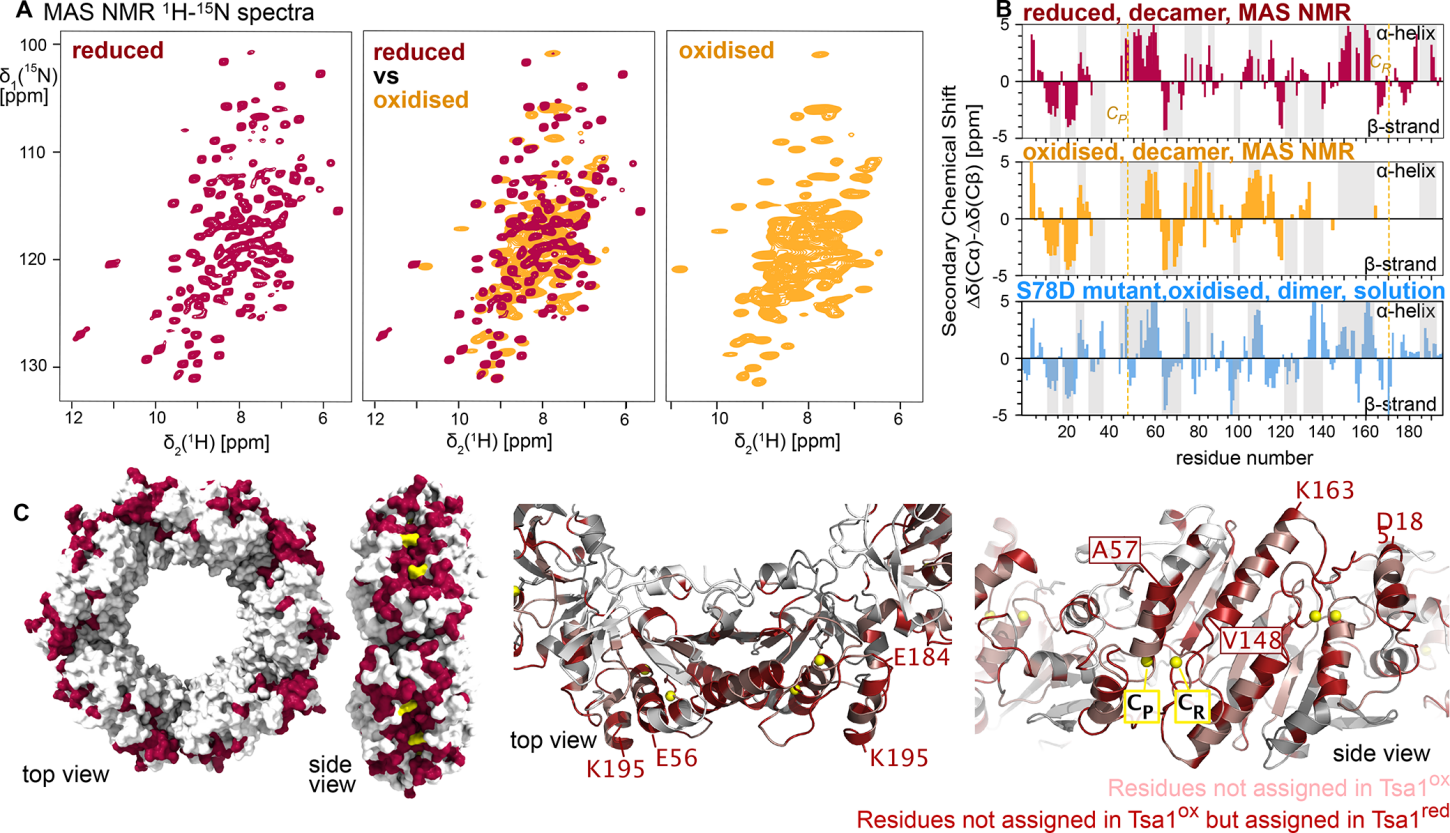
(A) ^1^H–^15^N MAS NMR spectra of the native oxidized state, Tsa1^ox^ (orange), and the reduced state, Tsa1^red^ (red), in the presence of reducing agent (TCEP) (^2^H, ^13^C,^15^N labeled protein, recorded at an MAS frequency of 55.5 kHz, 600 MHz ^1^H Larmor frequency; 1.3 mm rotor). (B) Secondary chemical shifts that report on residue-wise secondary structure propensities of the decameric Tsa1^ox^ and Tsa1^red^, and the dimeric S78D mutant (from solution NMR, see below). The secondary structures obtained from these data with the program TALOS-N^26^ are shown in Figure S6. Gray bars indicate the secondary-structure elements from the crystal structure of the decameric C47S mutant (3SBC).^94^ (C) Location of residues which cannot be detected in Tsa1^ox^. The Cys residues C_R_ and C_P_ are highlighted in yellow.

Magic-angle spinning (MAS) NMR avoids the signal loss/broadening induced by the slow tumbling in solution, by immobilizing the proteins:^24^ in the present case, we obtained MAS NMR samples by sedimenting (ultracentrifuging) decameric Tsa1 from a solution into an MAS rotor,^25^ thereby retaining a fully hydrated sample (approximately half of the rotor volume is aqueous buffer). Due to the absence of the overall tumbling in the sediment, MAS NMR spectra are not impacted by the particle size. We used MAS NMR to probe the structure and dynamics of the intact decameric assembly at ambient temperature. Under reducing conditions, i.e. in the presence of 5 mM TCEP in the solution, the MAS NMR spectra are of very high quality, as exemplified with a ^1^H–^15^N spectrum in Figure 1A. The reduced state stayed stable for more than one month in the MAS NMR rotor, as evidenced by unchanged spectra over at least this time period (possibly even longer; spectra of the oxidized state stayed unchanged for several months). Using a set of ^1^H-detected 3D H–N–C and H–N–N correlation spectra, we were able to assign the resonances of the majority (72%) of the backbone of this reduced state, Tsa1^red^. Example strip plots of the assignment are shown in Figure S4. The secondary structures, obtained from the chemical-shift assignment and TALOS-N,^26^ are in very good agreement with the crystal structure of Tsa1^C47S^ that cannot form the (Cys47-Cys170) disulfide bond (Figures 1B, S5 and S6).

### Extensive μs Motion in Decameric Tsa1 in the Oxidized State

The oxidized state, Tsa1^ox^, differs very markedly from the reduced one, as immediately visible from its ^1^H–^15^N spectrum (Figure 1A). The oxidized state is obtained after purification in nonreducing (native) conditions or, equally, by treatment with H_2_O_2_ (see Methods). Because formation of a disulfide bond links the catalytic cysteine, C_P_, of one subunit to the C_R_ of another subunit, the presence of a disulfide bond can readily be monitored by the appearance of dimeric species in sodium dodecyl sulfate–polyacrylamide gel electrophoresis (SDS-PAGE). Without any reducing treatment (but denatured by SDS which is expected to break noncovalent interactions within the decamer), Tsa1 indeed migrates as a dimer, demonstrating the presence of a disulfide bond; upon treatment with dithiothreitol (DTT) only monomeric species are observed (Figure S7). We have verified by NMR that the native state of a dimeric Tsa1 described below before and after H_2_O_2_ treatment are identical (Figure S8; done for a dimeric mutant, see below).

The MAS NMR line widths in the decameric Tsa1^ox^ are substantially larger than those of Tsa1^red^, and importantly, the number of observed cross-peaks is strongly reduced compared to Tsa1^red^: we observed ca. 92 ^1^H–^15^N cross peaks (combining information from 2D hNH and 3D hCANH/hCONH spectra, disregarding side chains peaks), for an expected 184 nonproline residues.

It is known that increased line width can be induced by insufficient hydration, particularly dramatic for lyophilized proteins.^27^ We can, however, safely rule out that the lower quality of the spectra of Tsa1^ox^ is the result of lower hydration levels: one-dimensional proton spectra show an intense water signal, similar to the one in Tsa1^red^ (Figure S9). As the protein is sedimented from a solution, it is indeed expected that the sediment contains well-hydrated protein.

The reduced number of amide signals might possibly also be the result of incomplete back-exchange of amides from ^2^H to ^1^H after the protein has been produced in *E. coli* in D_2_O (^2^H_2_O). However, we can also safely rule out this possibility for two reasons: first, the Tsa1^red^ sample has been obtained from Tsa1^ox^ by addition of reducing agent, and in Tsa1^red^ the amides are visible; second, comparison of solution-state NMR spectra of a dimeric mutant of Tsa1 produced in H_2_O does not show any additional peaks to samples produced in D_2_O, suggesting that amide reprotonation is not an issue (Figure S10).

We have considered the possibility that peaks are missing because they are highly flexible and dynamically disordered on ps–ns time scales. If this was the case, the dipolar coupling would be strongly reduced; as the dipolar coupling is the basis for coherence transfer in the above-mentioned experiments, such motion would indeed render the transfer inefficient, leading to very low signal. However, the J-coupling is independent of such motion, and J-coupling based experiments may allow transfer, but they require fast motion of significant amplitude in order to obtain sufficiently long coherence life times. (This requirement for fast large-amplitude motion is relaxed to some degree in the kind of deuterated proteins used here.) We observed for Tsa1^ox^ that J-coupling based experiments do not reveal any additional peaks (Figure S11). This observation allows us to exclude the hypothesis that fast (ps–ns) large-amplitude dynamics, reminiscent of random-coil behavior, causes the loss of those signals in dipolar-based experiments, which are observed in Tsa1^red^ but not in Tsa1^ox^.

We have used dipolar-coupling based 3D and 4D correlation experiments of ^1^H^N^, ^13^C, and ^15^N nuclei along the backbone, to obtain residue-specific assignments of the observed cross-peaks (see Methods). We were able to assign the vast majority of the detectable backbone resonances, which correspond to 88 residues along the sequence (45%). The near-complete assignment of the peaks observed in the spectrum allows identifying which parts of the structure are not detectable in this oxidized decameric state: the undetected parts cluster around the α-helix that harbors the peroxidatic Cys (residues 38–55), the internal β-strands β6 and β7 (residues 123–130 and 134–140), and the structurally adjacent large C-terminal part (residues 166–196) that comprises a long helix α6 (residues 149–165), a loop region that harbors the resolving-cysteine C_R_ and the C-terminal helix. When seen in the context of the decameric ring, the unobserved residues in Tsa1^ox^ are located on the outer rim of the ring (Figure 1C).

**Figure 2:**
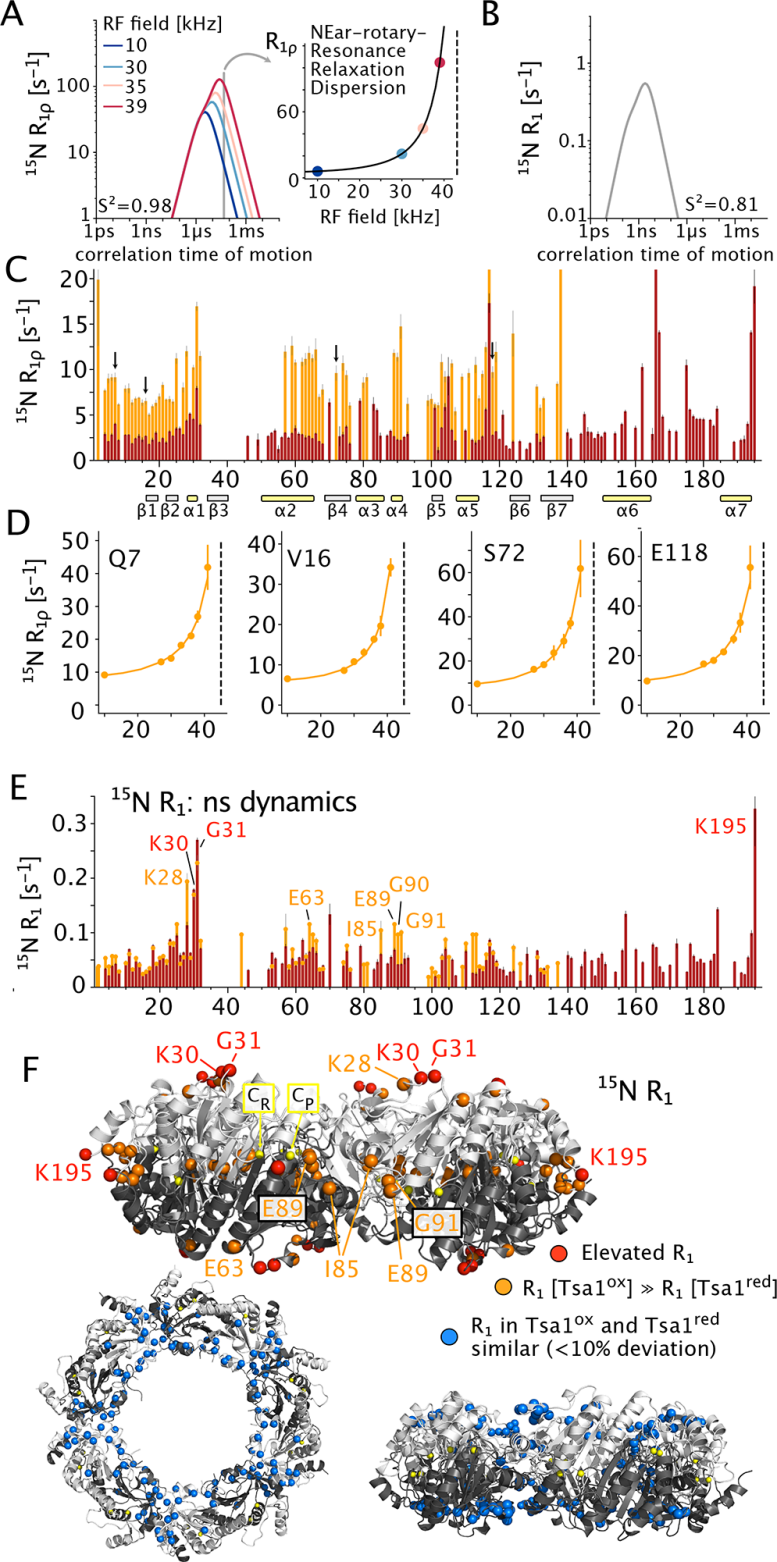
Quantitative dynamics measurements of Tsa1^ox^ (orange) and Tsa1^red^ (red). (A, B) Calculated ^15^N relaxation rate constants for motion of the H–N bond with an amplitude (1-S^2^, where S^2^ = 1 for a fully rigid site) and a correlation time as shown along the *x* axis. (A) ^15^N *R*_1ρ_ relaxation is most sensitive to motion on time scales from hundreds of ns to tens of ms. If the motion is slower than ca. 1 μs, *R*_1ρ_ depends on the spin-lock field strength, giving rise to NERRD dispersion profiles, i.e. a nonflat profile of *R*_1ρ_ vs spin-lock (insert). (B) Calculated longitudinal (*R*_1_) relaxation rate constants. (C) Experimental *R*_1ρ_ rate constants in Tsa1^ox^ (orange) and Tsa1^red^ (red), showing strongly enhanced relaxation in the oxidized state across the protein (55 kHz MAS, 14.1 T). Average values: *R*_1ρ_ (Tsa1^red^) = 3.9 ± 3.2 s^–1^; 3.3 s^–1^ for residues 1–140 and 5.6 s^–1^ for residues 140–C-terminus. *R*_1ρ_ (Tsa1^ox^) = 9.6 ± 4.0 s^–1^. Arrows indicate residues shown in (D). The rectangles at the bottom indicate the positions of β-strands (gray) and α-helices (yellow). (D) Nonflat NERRD profiles unambiguously reveal motion occurring on a μs time scale (45 kHz MAS, 14.1 T). (E) Longitudinal ^15^N relaxation in the two states showing that ns motion is similar for the two states for most residues (55 kHz MAS, 14.1 T). Residues with enhanced *R*_1_ relaxation in Tsa1^ox^ compared to Tsa1^red^ are highlighted orange. Residues with particularly high *R*_1_ are marked in red. Average values: *R*_1_ (Tsa1^red^) = 0.057 ± 0.041 s^–1^. (Tsa1^ox^) = 0.063 ± 0.037 s^–1^. (F) Visualization on the 3D structure of the residues with high *R*_1_ (red), residues with larger *R*_1_ in Tsa1^ox^ (orange), and residues with similar *R*_1_ in both states (blue).

To identify the origin of the peak loss, we measured ^15^N spin relaxation in Tsa1^ox^ and Tsa1^red^ by MAS NMR. Longitudinal relaxation (*R*_1_) is sensitive mostly to amplitudes and time scales of motions occurring on ps-ns time scales. Relaxation of ^15^N coherence in the presence of a spin-lock pulse (*R*_1ρ_) is mostly sensitive to motions on time scales of tens of nanoseconds to hundreds of μs.^28−34^Figure 2A, B shows calculated relaxation rate constants for motion occurring on different time scales. *R*_1ρ_ experiments can be applied at multiple radiofrequency (RF) spin-lock field strengths, and the dependence on the RF field strength reveals specifically μs–ms motion (Figure 2A, insert), an effect sometimes referred to as NEar Rotary-resonance Relaxation Dispersion (NERRD; see below).^30,35,36^ Thus, these different relaxation measurements allow the identification and quantitative interpretation of motional time scales and amplitudes in different time windows.

In the reduced state, Tsa1^red^, *R*_1ρ_ rate constants at 10 kHz spin-lock amplitude are ca. 2.9 s^–1^ (median), and longitudinal relaxation rate constants (^15^N *R*_1_) are ca. 0.05 s^–1^. These are typical values for compact folded proteins without large-scale ns−μs motion.^37−43^ Residues with higher-than-average relaxation rate constants include K30, G31, E117, V167, which are all located in loop regions, as well as the very C-terminal residues. The larger-amplitude motions reflected by these data are also typical for loops and termini. Overall, the ^15^N relaxation data show that Tsa1^red^ is rather rigid on the ps–ns time scales (sensed mostly by *R*_1_) and ns−μs (sensed mostly by *R*_1ρ_) .

The oxidized state, Tsa1^ox^, differs strongly from its reduced counterpart, particularly with respect to transverse relaxation: the average *R*_1ρ_ is ca. 3-fold higher under the same conditions as used for the reduced sample. This finding unambiguously shows the presence of motion that is either of larger amplitude (on the ns−μs time scale) or shifted toward the μs range, where *R*_1ρ_ is highest (Figure 2A). Longitudinal relaxation, reflecting faster motion, is more similar in the two states. However, the residues for which significantly faster *R*_1_ relaxation is observed are located in the vicinity of the two cysteines, suggesting that disulfide bond formation enhances not only μs motion but also faster (ns) dynamics (Figure 2F). Residues further away from the disulfide-bonded parts tend to have more similar *R*_1_ values (blue in Figure 2F). The overall enhanced transverse relaxation in Tsa1^ox^ also provides a rationale why J-coupling based experiments do not provide additional (but rather much less) signal, as discussed above: enhanced transverse relaxation leads to rapid loss of signal in INEPT experiments.

To obtain more precise information about this motion, we performed ^15^N *R*_1ρ_ NEar-rotary Resonance Relaxation Dispersion (NERRD) experiments; i.e., we measured *R*_1ρ_ rate constants as a function of the applied spin-lock field strength, approaching the regime where the spin-lock field strength (nutation frequency ν_RF_) approaches the MAS frequency ν_MAS_. Nonflat NERRD profiles directly point to motion on time scales of μs (see Figure 2A). The underlying mechanism is the fluctuation of the H–N bond orientation (dipolar coupling and chemical-shift anisotropy, CSA).^28^ In Tsa1^ox^ we find strong NERRD effects for all observed residues (Figures 2D and S12).

**Figure 3:**
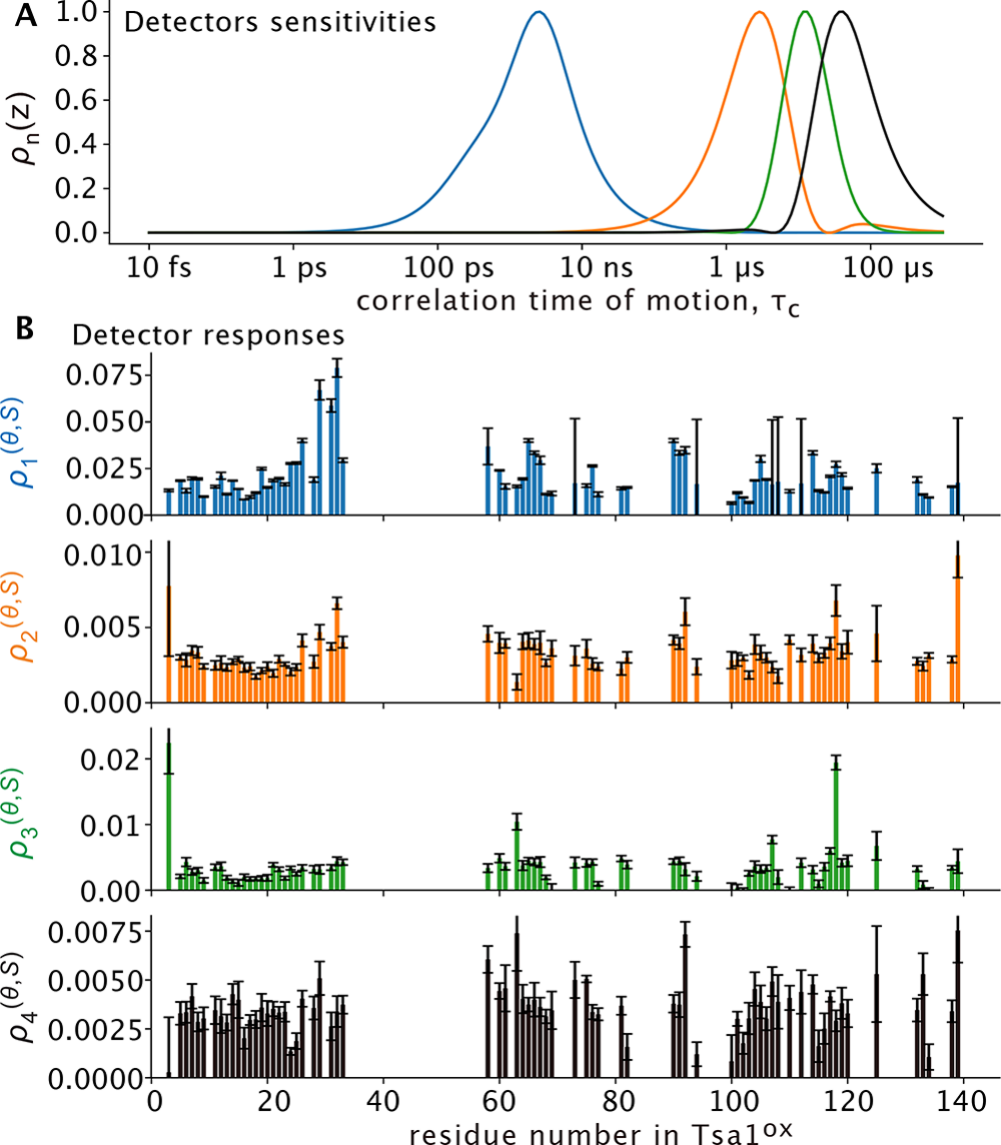
Distribution of dynamics in Tsa1^ox^ from MAS NMR relaxation data, as obtained from the detectors analysis^45^ (github.com/alsinmr/pyDR). (A) Sensitivity of the four detectors, ρ_n_, to different time scales of motion. (B) Detector responses of these four detectors, reflecting motional amplitudes in the time windows depicted in (A). All *R*_1ρ_ data (see Figure S12) and the *R*_1_ data of Tsa1^ox^ were used for this fit.

The relaxation rate constants for the different experiments (*R*_1_ and *R*_1ρ_ at different RF field strengths) are the result of motions occurring with a distribution of amplitudes (1 – *S*^2^) and time scales (τ_c_).^44^ To gain more quantitative insight, we jointly fitted eight relaxation rate constants (seven *R*_1ρ_ and one *R*_1_) with the detectors approach.^45^ This formalism reports on the amplitudes of motions in different time windows (named detectors). Rather than fitting μs dynamics as a single process at one time scale, as commonly done in solution and solids,^46^ the spin relaxation is modeled as resulting from combined dynamical processes with correlation times across the ns–ms range, assuming a distribution of correlation times θ(*z*), given on a logarithmic scale, where *z* = log_10_ (τ_c_/s). The detectors, which report on the distribution of motions over the range of correlation time, are shown in Figure 3A, and the responses of these detectors (reporting on the respective amplitudes) for Tsa1^ox^ are shown in panel B. This analysis points, on the one hand, to enhanced ns motion of the loop region comprising K30 and G31 (indicated also by the *R*_1_ data of Figure 2E, F). On the other hand, μs motion is found for most residues across the protein in Tsa1^ox^, mirroring the generally high levels of *R*_1ρ_ and the nonflat NERRD profiles. The last observed residue, T138, as well as residues around the nonassigned part (residues 34–56), have particularly enhanced μs mobility; this is also evident from the data at 10 kHz RF field strength (Figure 2C). This finding supports the notion that the parts that are not visible in Tsa1^ox^ are broadened due to μs motion of even larger amplitude which, thus, have even larger transverse relaxation.

We have furthermore applied Bloch–McConnell relaxation dispersion (BMRD) experiments, which detect μs–ms exchange processes based on chemical-shift fluctuations (rather than fluctuations of the dipolar coupling and CSA, which are the basis of NERRD): in BMRD, μs–ms modulation of the chemical shift leads to enhanced transverse relaxation, which can be quenched by a spin-lock that is sufficiently strong; thus, a decrease of *R*_1ρ_ rate constants when increasing the RF field strength from ca. 2 to above 10 kHz reveals chemical-shift fluctuations on μs–ms time scales.^30,35,46,47^ Below ca. 2 kHz, insufficiently suppressed dipolar dephasing may lead to increased *R*_1ρ_.^35^ (Because the NERRD effects in Tsa1^ox^ are large and extend even to low RF field strengths where BMRD effects are expected, we corrected the BMRD data by subtracting the dipolar and CSA contributions; see Methods and Figure S13.)

**Figure 4:**
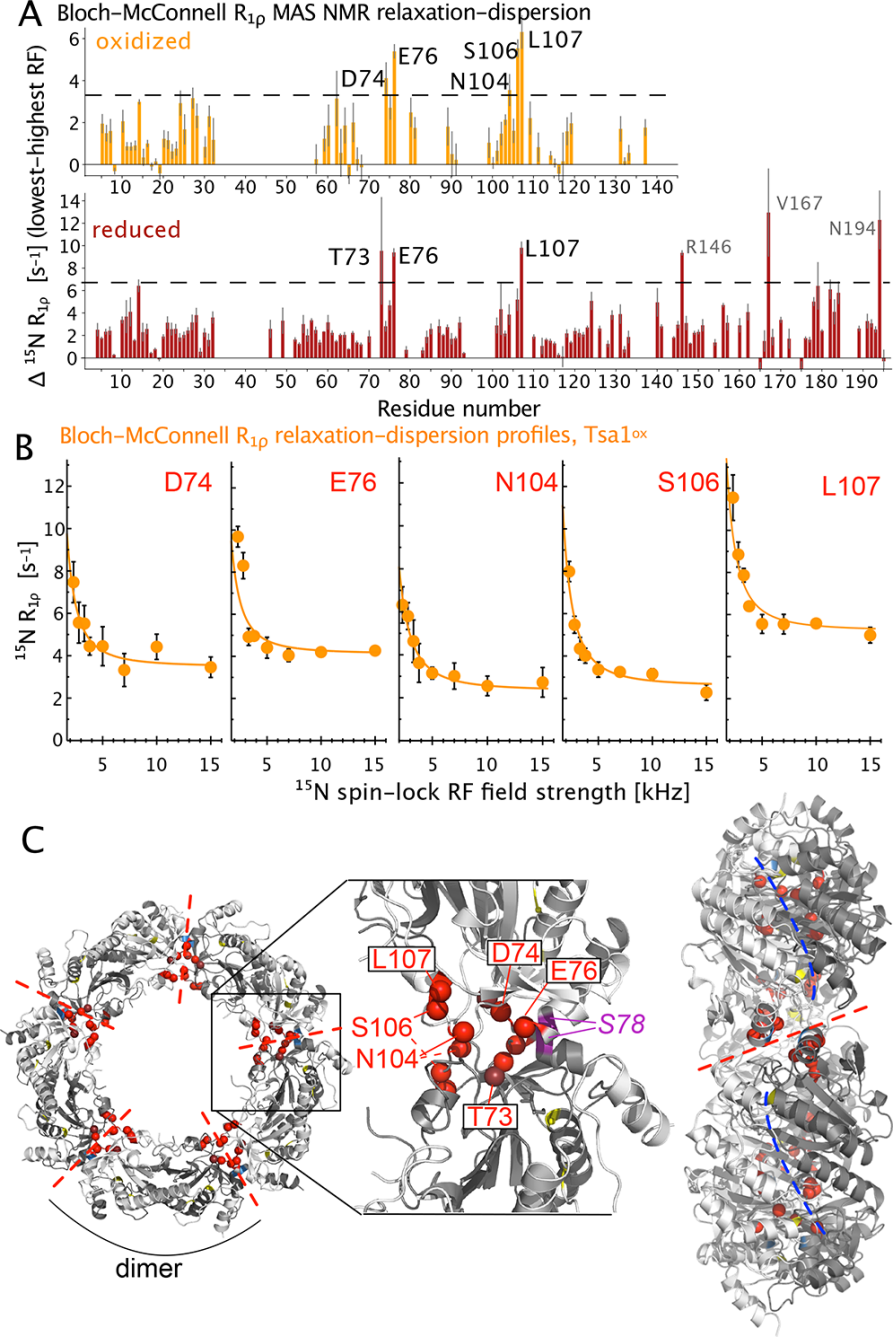
Bloch–McConnell *R*_1ρ_ MAS NMR relaxation-dispersion (BMRD) data of Tsa1^ox^ (orange) and Tsa1^red^ (red), obtained at 55 kHz MAS frequency, 14.1 T. (A) Difference of *R*_1ρ_ obtained at low RF field strength (2.3 kHz) minus *R*_1ρ_ at high RF field strength (15 kHz for Tsa1^ox^; 10 kHz for Tsa1^red^). The horizontal line indicates the 3-fold standard deviation over all residues. (B) BMRD profiles for Tsa1^ox^ for the residues with the largest dispersions. (BMRD curves of all residues are shown in Figure S14.) The *R*_1ρ_ rate constants have been corrected for the NERRD effect, by back-calculating and subtracting the rate constants based on detectors fits^45^ of the NERRD data of Figure 2D, as outlined in Figure S13. Residue-wise frequency offsets have been corrected with *R*_1_ data, as commonly done.^46^ Solid lines show a joint fit of the 5 residues (*k*_ex_ = 1100 s^–1^) to a two-state exchange model. (C) Location of the residues with the largest BMRD effects in the decameric Tsa1 structure (PDB 3SBC).

Figure 4 shows that residues with significant BMRD effects are located at the interface of dimeric building blocks. Interestingly, similar effects are found for both the oxidized and reduced species. We ascribe this process to some flexibility within the dimer-to-dimer interface, which prompted us to investigate more closely the dimeric state and the oligomerization.

### Solution-NMR of dimeric Tsa1 reveals disorder around the cysteines

To understand the effects of decamer formation from dimers on structural and dynamical properties, we searched for ways of stabilizing the dimer. At ambient conditions Tsa1 predominantly exists in the decameric state, where solution-NMR spectra are of poor quality (in Figures S2, S3, S15). We followed how temperature changes the oligomerization state by performing methyl-detected diffusion-ordered NMR spectroscopy (DOSY) in solution using a deuterated, Ile/Leu/Val methyl-labeled sample. Because of the high intrinsic sensitivity of methyl groups, together with the methyl-TROSY effect,^48^ the signals of methyl groups are detectable even in the 216 kDa-large decameric Tsa1 assembly (Figure S15), which allowed us to quantify the diffusion coefficient. Temperature-dependent DOSY data reveal the transition from the decameric to a dimeric state with a midpoint at ca. 315 K above which Tsa1 is predominantly dimeric (Figure S16).

However, prolonged experiments at such high temperatures are not compatible with the sample integrity, which prompted us to insert a mutation in the dimer–dimer interface that disrupts decamer formation also at low temperature. The S78D mutant, identified before,^49^ appears entirely dimeric as seen by size-exclusion chromatography (Figure S2C). Note that S78 is located close to the cluster of residues for which we detected μs motion in the decameric state by BMRD experiments (Figure 4C). In contrast to the wild-type protein, Tsa1^S78D^ yields excellent solution-NMR spectra of both backbone and methyl groups already at 298 K (Figures S3 and S15). We assigned the backbone resonances as well as a large extent of the methyl resonances of Ile (δ1), Val (γ1 and γ2), and Leu (δ1 and δ2) of the oxidized state, which corresponds to the form obtained after purification from *E. coli* (Figure S8).

The chemical-shift derived secondary structure propensity of the S78D dimer is largely in agreement with the crystal structure of Tsa1 and with the chemical-shift derived secondary structures of the decameric state by MAS NMR, but there are notable exceptions (Figure 1B). Importantly, the part that carries the peroxidatic Cys-47 (helix α2) does not have chemical shifts of a stable α -helix, unlike in the decameric state, which shows that this helix is marginally stable in the Tsa1^S78D^ dimer.

**Figure 5:**
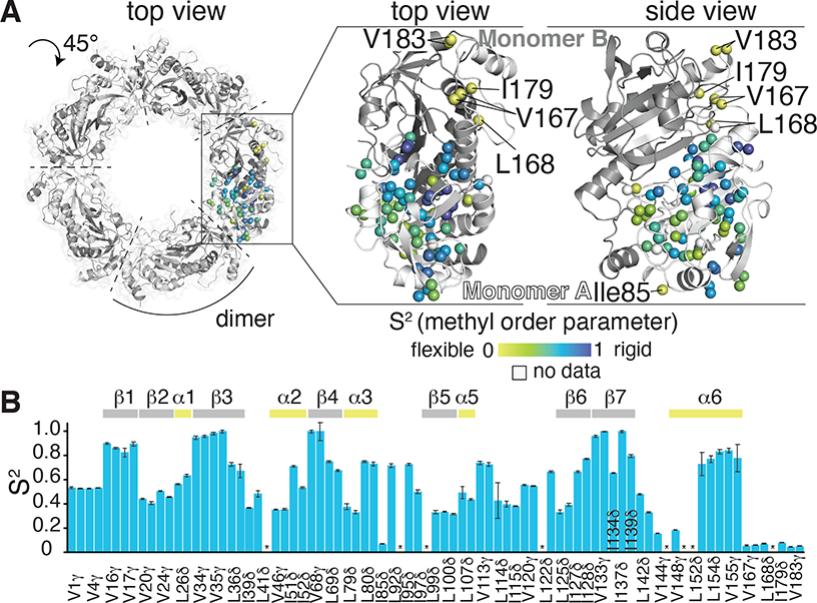
Dynamics of the dimeric state, probed by solution-NMR in Tsa1^S78D^ of Ile, Leu, and Val methyl groups at 37 °C. (A) Methyl order parameters (*S*^2^) plotted on the structure of one Tsa1 monomer as indicated by the yellow to blue gradient. (The decameric state is shown here for better reference to the MAS NMR data, but the data were obtained on the dimeric state.) The top view was rotated by 45° along the central decamer axis compared to Figure 1C. Residues in the C-terminal part and the surface exposed Ile85, showing the highest degree of dynamics, are annotated. (B) *S*^2^ values plotted against the sequence. Tsa1 secondary structure elements are indicated on the top. Residues for which no data could be obtained are marked with *.

To gain direct insights into the dynamics, we have performed backbone ^15^N amide and Ile, Leu, Val methyl ^1^H–^13^C triple-quantum relaxation experiments (Figures S17 and 5). Backbone ^15^N transverse relaxation rate constants (*R*_1ρ_) are ca. 2–3-fold reduced for residues located C-terminal to residue 180, including the part the forms helix α7 in the decamer. Moreover, the methyl order parameters of residues in this part (V167, L168, I179, V183) are low (*S*^2^ ∼ 0.1), much lower than for the majority of the protein, providing independent evidence that the C-terminal part has significant disorder in the dimeric, oxidized Tsa1^S78D^ (Figure 5). These findings show that the disorder in this part of the protein exists also in the dimer.

### Structural Frustration around the Dynamic Disulfide Part

Collectively, our NMR data have revealed that the formation of the disulfide bond induces dynamic disorder. As a consequence, signals in MAS NMR spectra of the oxidized state are broadened beyond detection, and we find broadening and disorder (in particular of the peroxidatic Cys–(C_P_)-carrying helix and the C-terminal ca. 20 residues) also in the dimer. One may expect that the μs motion that we have revealed here is reflected in structural heterogeneity when viewed by X-ray crystallography. This hypothesis can be tested for the case of the human homologue (PRDX2), for which structures of reduced (C_P_–SH), hyperoxidised (C_P_–SO_2_H),^50^ and oxidized (C_P_–S–S–C_R_)^7^ decameric states have been determined. For the reduced and hyperoxidised states of PRDX2, as well as for reduced-state-mimicking Tsa1^C47S^, essentially the entire protein has been modeled at ca. 1.7 to 2.3 Å resolution, and with only modestly increased B-factors toward the very C-terminal residues and low B-factors for the C_P_-carrying helix (Supplementary Figure S18). In the oxidized state, however, the entire C-terminal part, comprising the two last helices and the connecting loop, as well as the helix containing the C_P_ have elevated B-factors, and ca. 40 residues have not been modeled in the crystallographic model; moreover, superposition of the ten subunits of the decamer reveals significant structural heterogeneity (Figure S18E).

**Figure 6:**
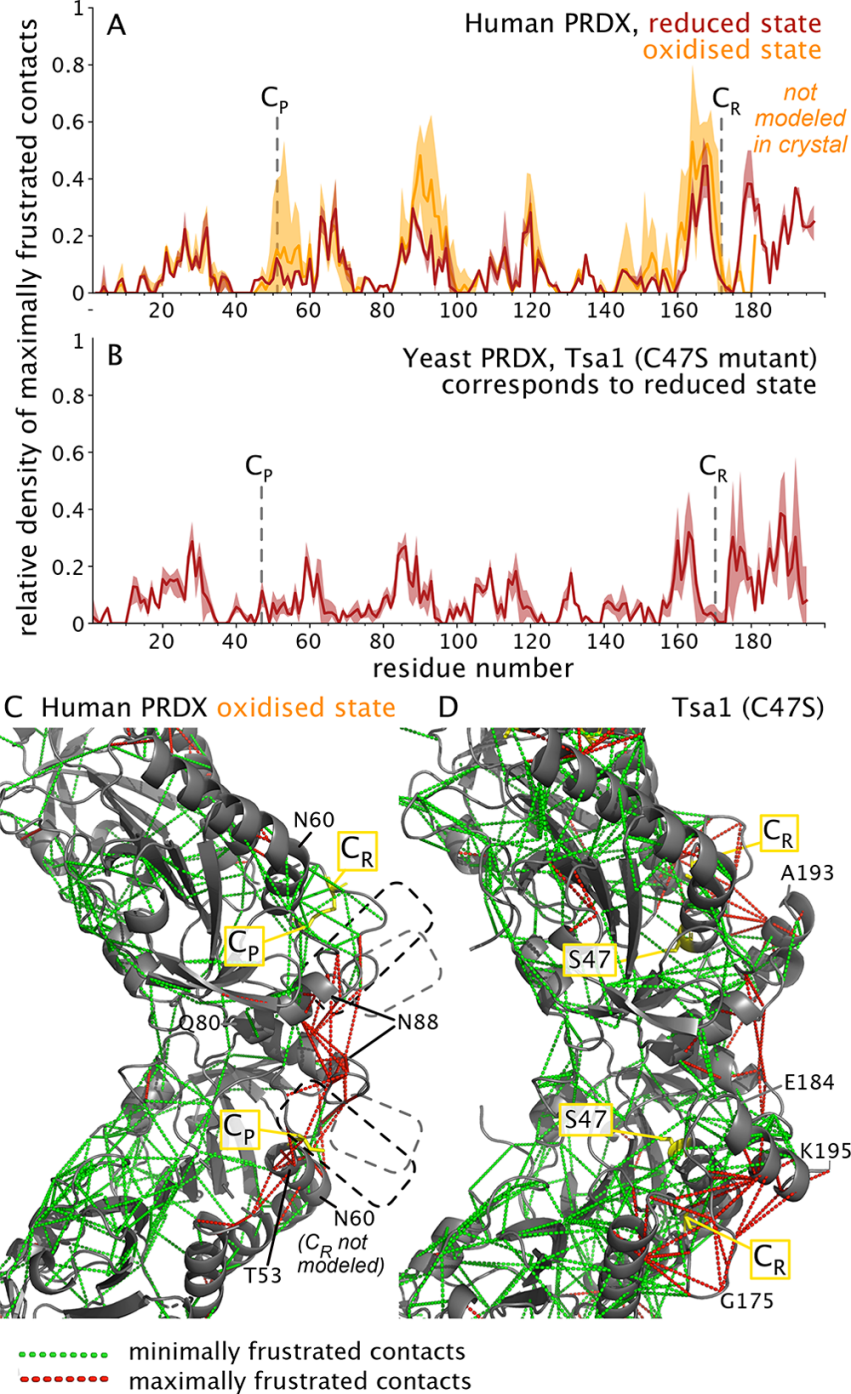
Structurally frustrated parts of PRDX coincide with the outer rim where extensive line broadening is observed. (A, B) Density of maximally frustrated contacts in human PRDX (A) in the oxidized and reduced states (PDB 5IJT(7) and 7KIZ,^50^ respectively) and the Tsa1 mutant C47S that mimicks the reduced state (PDB 3SBC(94)), plotted along the sequence position. The data have been obtained with the Frustratometer Web server.^95,96^ The solid line shows the average value over all available data in the decamer, and the shaded area denotes the range between the highest and lowest value found. (C, D) Clusters of maximally and minimally frustrated contacts, shown by red and green dashed lines, respectively, on the structures of human PRDX^ox^ (C) and Tsa1^C47S^. The data have been obtained in the same Frustratometer analysis as in (A, B). In the structure of PRDX^ox^, some parts have not been modeled (located in regions indicated by dashed boxes; see also in Figure S18), including some of the C_R_ cysteines. Additional data for another homologue are shown in Figure S19.

We propose that this dynamic disorder induced by the C_P_–S–S–C_R_ bond formation is due to conflicting structural constraints within the protein, also denoted as structural frustration,^22^ which arises when multiple energetically favorable interactions cannot be simultaneously fulfilled. As a consequence of structural frustration, the protein explores a broad shallow conformational landscape on longer time scales, rather than populating a narrow range of conformations with low-amplitude (fast) fluctuations. To explore this idea we have used the available crystal structures and determined the structural frustration for the reduced and oxidized states of PRDX2 (Figure 6A) and also of the reduced-mimicking state of Tsa1 (C47S mutant; no crystal structure of oxidized Tsa1 is available; Figure 6B). These data show a cluster of structurally frustrated sites at the outer rim of the decameric ring (ca. residues 165 to the C-terminus) already in the reduced state (red in Figure 6A and right side in panel B). The oxidized state has a substantially increased number of maximally frustrated sites which cluster in particular in the parts comprising the two cysteines and the structurally neighboring part of residues ca. 80–100. As noted, a large C-terminal stretch that forms an α-helix in the reduced state is not observed in the oxidized state. Taken together, in light of the strong correlation of dynamic (μs motions, blurred electron density) and structurally frustrated sites, we propose that structural frustration induced by disulfide-bond formation is at the core of the functional cycle of PRDXs.

## Conclusion

**Figure 7:**
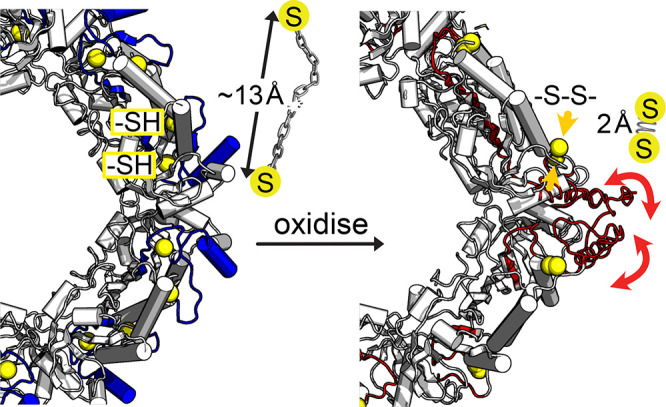
Sketch illustrating the proposed effect of disulfide bond formation in Tsa1. In the reduced state (left) the distance between C_P_ and C_R_ is large (ca. 13 Å). Formation of the disulfide bond (right) induces a geometric constraint on the rest of the structure, which leads to structural frustration and μs dynamics. The figure on the right shows superimposed three conformations of the C-terminal part (from residue 175 on), which have been built with CHARMM-GUI and manual modification of dihedral angles; the structures are intended to be for illustration purposes, but do not necessarily represent physically realistic states.

Disulfide bond formation is generally associated with an increased stability of a folded state.^51^ Contrary to this view, our quantitative dynamics investigations have shown the appearance of collective μs motions upon formation of the key catalytic disulfide bond in a peroxiredoxin. Our data mirror the lack of visible density in crystallographic structures of the decameric assembly. Our solution-NMR data, using a mutant that stabilizes the dimer, point to dynamic disorder already in the dimeric state. Our data reveal a link between slow (μs) dynamics, structural frustration, and disulfide-bond formation. We ascribe the appearance of dynamics to the structural strain imposed by the disulfide onto the rest of the protein (see Figure 7). The link between disulfide-bond formation and structural frustration found here for Tsa1 may well be more general; indeed, a recent study reports disulfide-bond induced dynamics in the oxidoreductase DsbD.^52^ Although the extensive dynamics hamper the actual visualization of the structural ensemble at the atomic level, we propose that the disulfide-induced dynamics likely leads to exposure of hydrophobic patches and an increase of the conformational entropy. Both of these effects have been ascribed to chaperone function: the increased entropy would lower the free-energy penalty associated with binding a disordered protein to the chaperone, and the exposure of hydrophobic patches is, generally, a key to chaperone mechanisms.^53,54^ Ongoing studies assess the link of the observed structural frustration and chaperone activity of PRDXs.

## Methods

### Cloning

The plasmid harboring wild-type Tsa1 (pET19b-Tsa1; corresponding to UniProt entry P34760) was originally obtained with an amino-terminal deca-histidine-tag in a codon-optimized manner from GenScript (kind gift from P.O. Widlund/T. Nyström). A S78D single point mutation was introduced into the Tsa1 plasmid by standard methods using the following forward 5′ GGT CCA CGC CAG CAG GTC GTA TTC GCT ATC GGT G 3′ and reverse 5′ CAC CGA TAG CGA ATA CGA CCT GCT GGC GTG GAC C 3′ primers. Primers were purchased from Eurofins.

### Protein Expression and Purification

Tsa1 wild-type or Tsa1^S78D^ plasmids were chemically transformed into *E. coli* BL21(DE3) competent cells. The cell culture was initiated by inoculation of a single colony into a 20 mL preculture of LB medium supplemented with 100 μg/mL Ampicillin, and subsequently incubated at 37 °C overnight. This preculture was used to inoculate 1 L of LB culture medium supplemented with 100 μg/mL Ampicillin, and cells were grown until an OD_600_ ∼ 0.6 was reached. Expression was induced by the addition of 0.3 mM IPTG, and cells were grown for an additional 3 h at 37 °C. Cells were harvested by centrifugation at 6,000*g* for 25 min at 4 °C, subsequently resuspended in lysis buffer (20 mM Tris pH 8, 250 mM NaCl, 5 mM imidazole), and flash-frozen in liquid nitrogen until purification. After the cells were thawed, one cOmplete EDTA-free Protease Inhibitor Cocktail tablet (Roche), HL-SAN DNase I (ArcticZymes), and 1 mM MgCl_2_ were added to the lysate. Cells were lysed by 4 passes through an Avestin Emulsiflex C3 (pressure between 20,000–25,000 psi) and centrifuged at 24,000*g* for 45 min at 4 °C. The cleared supernatant was applied to a Ni^2+^–HisTrap column (GE Healthcare) and eluted with a 3-step imidazole gradient in lysis buffer ranging from 150 mM to 500 mM and finally 1 M imidazole. Tsa1 typically eluted within the 500 mM imidazole fraction. The fractions containing Tsa1 were dialyzed against lysis buffer overnight at 4 °C, concentrated by ultracentrifugation (Vivaspin concentrators, Sartorius), and applied to a gel filtration column (Superdex S200 increase, GE Healthcare) equilibrated with NMR buffer (typically 50 mM potassium-phosphate, pH 7.4, 50 mM KCl or PBS pH 7.4). Fractions of the same oligomeric state were concentrated, and the concentration of the sample was quantified by measuring the optical density (OD_280_) based on the theoretical molar extinction coefficient of 24,500 M^–1^ cm^–1^.

### Isotope Labeling

Isotope labeled proteins were expressed either in H_2_O or D_2_O based M9 minimal media supplemented with the desired isotopes.^55^ For the uniformly labeled [U–^15^N] and [U–^15^N, ^13^C] proteins, (^15^NH_4_)Cl and D-(^13^C)-glucose were used, whereas for [U–^2^H,^15^N,^13^C] labeling, D_2_O-based M9 medium supplemented with (^15^NH_4_)Cl and D-(^2^H,^13^C)-glucose were used. For production of specific methyl-group labeled Tsa1, D_2_O-based M9 medium with D-(^2^H, ^12^C)-glucose and (^15^NH_4_)Cl was used, and deuterated biosynthetic precursors (keto-acids, see below) with specific ^13^CH_3_ labeling were added to the culture 1 h prior to induction at OD_280_ around 0.5: for labeling of isoleucine, leucine and valine (ILV) methyl groups, 85 mg/L of α-ketoisovalerate-(3-methyl-^13^C),4-^13^C, 3-d and 50 mg/L of [^2^H], 3,3-[^13^CH_3_]-ketobutyrate were added to the medium resulting in [U–^2^H,^15^N, Ile-δ 1-^13^CH_3_, Leu/Val-^13^CH_3_] labeled Tsa1 and Tsa1^S78D^.^56^ The stereospecific labeling of valine methyl groups only was achieved by adding 85 mg/L of α-ketoisovalerate-(3-methyl-^13^C),4-^13^C, 3-d and 40 mg/L of l-leucine-d10, to produce [U–^2^H,^15^N, Val-^13^CH_3_] labeled Tsa1^S78D^ sample.^57^ All stable isotopes were purchased from Merck.

### Solution NMR Spectroscopy

NMR measurements were performed on a Bruker Avance III HD 700 or 800 MHz spectrometer, using Topspin 3.5 software and equipped with either TCI or TXO cryogenically cooled triple resonance probes. All experiments were performed either in 50 mM KPi, 50 mM KCl, pH 7.4 buffer or PBS pH 7.4 at the indicated temperatures. For the sequence specific backbone resonance assignments of [U–^2^H,^15^N,^13^C] Tsa1^S78D^ the following TROSY-type experiments were recorded at 37 °C: 2D [^15^N, ^1^H]-TROSY,^23^ 3D trHNCO, 3D trHNCA, 3D trHNCACB, 3D trHNCOCACB.^58^ Complementary sequence specific backbone and side-chain assignments of [U–^15^N, ^13^C ] Tsa1^S78D^ were recorded: BEST-type triple resonance experiments 3D BT-HNCA+, 3D BT-HNCO,^59^ BT-HNCACB+,^59,60^ and CBCA(CO)NH.^61^ Aliphatic side-chain resonance assignment for [U–^15^N, ^13^C] Tsa1^S78D^ was performed based on 2D ^1^H–^13^C HMQC spectra with/without the constant time version, as well as 3D HBHA(CO)NH, and HCCH-TOCSY-experiments.^61^ To confirm methyl group assignments on ILV-samples 3D ^13^C methyl-SOFAST-NOESY experiments^62,63^ with mixing times of 50 and 600 ms as well as a 3D HMBC-HMQC spectrum^64^ were measured in 99.8% D_2_O-based NMR buffer. For quantitative analysis of signal intensities, the amplitudes were corrected by differences in the ^1^H-90° pulse length, the number of scans, and the dilution factor.^65^ Further, a weighting function with weights 1–2–1 for residues (i–1)–i–(i+1) was applied to the raw data.^66^ NMR data were processed with a combination of NMRPipe^67^ and mddNMR2.6,^68^ and analyzed using CARA.^69^ Secondary chemical shifts were calculated relative to the random coil values calculated by the POTENCI program.^70^ Methyl group assignment was performed using the MAGIC algorithm^71^ and confirmed by a combination of manual analysis of the side-chain TOCSY, HMBC-HMQC, and NOESY data sets. This approach yielded the following degree of assignment (∼92%): Ile δ1 (13/13), Leu δ1, δ2 (30/34), and Val γ1, γ2 (34/36).

Translational diffusion coefficients were measured by recording a series of 1D ^13^C-edited DOSY spectra at different temperatures ranging from 25 to 60 °C, using a pulse scheme (^13^C -edited BPP-LED^72^) that is similar to a ^15^N-edited BPP-LED experiment with ^15^N and ^13^C pulses interchanged.^73^ The gradient duration δ was adapted to 3.2 ms instead of 4.8 ms used in the ^15^N-filtered version, and a diffusion delay T of 400 ms and a τ of 0.1 ms were used. The strength of the encoding/decoding was increased stepwise. The resulting ^1^H signal was integrated over the methyl ^1^H frequency range to obtain intensities as a function of encoding/decoding gradient strength. In addition, 1D ^1^H diffusion experiment (DOSY) with ^13^C filter and decoupling were recorded at the same temperatures. To adjust for the temperature-dependent changes in viscosity of the buffer, the obtained translational diffusion coefficient was adjusted on the basis of temperature-dependent viscosity values for D_2_O-based buffers over the used temperature range.^74^ DOSY data were compared to previously reported data to obtain a quantitative benchmark,^75−77^ as described in Figure S16. Structure-based diffusion coefficients (Figure S16) were obtained with HYDROPRO.^78^

### Magic-Angle Spinning NMR Sample Preparation

2.5 mg of [U–^2^H,^15^N,^13^C] Tsa1^WT^ protein sample in 50 mM KPi, 50 mM KCl, pH 7.4 buffer were thawed at room temperature and sedimented into a 1.3 mm rotor using ultracentrifugation at 50,000*g* overnight at 6 °C, using an in-house-built device for ultracentrifuges.

### Magic-Angle Spinning NMR Spectroscopy

MAS NMR experiments were recorded on a Bruker Avance-III spectrometer at a ^1^H Larmor frequency of 600 MHz, equipped with a 1.3 mm probe tuned to ^1^H, ^13^C, ^15^N, and ^2^H frequencies. Sequence-specific backbone resonance assignments of [U–^2^H,^15^N,^13^C] Tsa1^WT^ have been recorded at ca. 25 °C effective sample temperature at 55 kHz magic angle spinning (MAS) by the following ^1^H detection experiments: 2D hNH, 3D hCONH, 3D hCOcaNH, 3D hCANH, 3D hCAcoNH. 3D hCACBcoNH, 3D hCACBcacoNH, 3D hcaCBCAcoNH, 4D hCACONH, 4D hCOCANH and 4D hCACBcaNH and 3D hNcocaNH and 3D hNcacoNH experiments. The experiments use cross-polarization (CP) transfer steps for all heteronuclear transfers; for CA-CO transfer a BSH–CP was used;^79^ CA-CB out-and-back transfers used 6.5 ms-long INEPT transfers. All experiments were used as implemented in the NMRlib library.^80^ In 3D experiments the ^15^N dimension was sampled up to a 16.4 ms evolution time (40 ppm spectral width; 80 points). In the hCANH experiment, the CA dimension was sampled to 6.6 ms (40 ppm; 80 points; 24 scans per increment); in the hCONH experiment, the CO dimension was sampled to 12.3 ms (15 ppm; 56 points; 40 scans); in the hcaCBcaNH experiment, the CB dimension was sampled to 6.8 ms (70 ppm; 144 points) and in the hcaCBcacoNH experiment to 6.0 ms (70 ppm; 128 points; 24 scans); in the 3D N–N–H experiments the two N dimensions were both sampled to 14.8 ms (hNcocaNH; 40 ppm; 72 points; 48 scans) or 15.2 ms (hNcacoNH; 40 ppm; 74 points; 80 scans). The acquisition parameters for the 4D experiments were set as follows. 4D hCACONH: CO (7.1 ms; 13 ppm; 28 points); N (12.3 ms; 36 ppm; 54 points); CA (5.1 ms; 40 ppm; 62 points). hCOCANH: CA (5.2 ms; 38 ppm; 60 points); N (13.2 ms; 36 ppm; 58 points); CO (7.1 ms; 13 ppm; 28 points). hcaCBCANH: CA (4.0 ms; 30 ppm; 36 points); N (9.1 ms; 36 ppm; 40 points); CB (3.4 ms; 70 ppm; 72 points). The number of scans per increment was 8 in the three 4Ds, and the grids were uniformly sampled; nonuniform sampling could have been used to reduce acquisition time, at some expense of sensitivity. The use of high-dimensional assignment experiments has been demonstrated by several groups.^81−85^ The 4D data have been very valuable for the assignment to discriminate different assignment possibilities of the 3D-based assignment strategy. The NMR data were processed with NMRPipe^67^ and subsequently analyzed with CcpNmr Analysis 3.0.1.^86^

### NMR Backbone and Side Chain Dynamics

For the analysis of the dynamic properties of Tsa1^S78D^, the following relaxation experiments were measured: *R*_1_(^15^N),^87^^15^N(^1^H)-NOE (hetNOE),^87^*R*_1__ρ_(^15^N),^88^ and TROSY for rotational correlation times (TRACT).^89^ Nonlinear least-squares fits of relaxation data were done with NMRFAM-Sparky 1.47.^90^ Side-chain dynamics experiments were performed on a [U–^2^H, ^15^N, Ile-δ1-^13^CH_3_, Leu-, Val-^13^CH_3_] labeled Tsa1^S78D^ sample at 37 °C in 99.9% D_2_O based NMR buffer. Side chain methyl order parameters (S^2^) were determined by a relaxation-violated coherence-transfer triple-quantum experiment.^91^ The buildup of triple-quantum (3Q) coherence was monitored in a series of ^1^H–^13^C experiments at different delay times (13 values from 1 to 35 ms). The decay of single-quantum (SQ) coherence was followed in a set of reference experiments at the same delays. Ratios of the peak intensities of these two sets of experiments were fitted to obtain site-specific order parameter S^2^ using the separately determined (TRACT-approach) overall correlation time τ_c_ in 100% D_2_O of 27 ns.

For the analysis of the dynamic properties of Tsa1^WT^, ^15^N-*R*_1_ and ^15^N-R_1ρ_ relaxation experiments were measured by MAS NMR on the [U–^2^H,^15^N,^13^C] Tsa1^WT^ sample. The experiments were based on hNH 2D experiments with CP transfer.^40^^15^N-*R*_1_ was measured at a MAS frequency of 55 kHz, and relaxation delays of 0.1 to 15 s. A series of ^15^N-R_1ρ_ experiments were collected at delay times ranging from 1 to 200 ms at ^15^N spin-lock radio frequency field strengths from 2.3 to 15 kHz, resulting in a Bloch–McConnell-type relaxation-dispersion experiment.^35^ This approach monitors microsecond–millisecond dynamics by the chemical-shift fluctuations. NEar-Rotary-Resonance relaxation Dispersion (NERRD) ^15^N-R_1ρ_ experiments were recorded at a MAS frequency of 45 kHz, which allows approaching the rotary-resonance condition (spin-lock RF field equals MAS frequency) without requiring extensively high RF fields. A series of 2D spectra with spin-lock durations ranging from 1 to 50 ms was recorded at RF field strengths from 10 to 41 kHz. This experiment detects μs–ms motion involving bond reorientation.^35,36^ All experiments are implemented in NMRlib.^80^

The analysis of the NERRD data was performed using the pyDIFRATE program, a python-based tool for detectors analysis (github.com/alsinmr/pyDR).^45^ We used either three or four detectors, both of which give similar results (see Figure 3).

Given the large NERRD effects for almost all residues, which extends to even below 10 kHz, the analysis of Bloch–McConnell relaxation-dispersion (BMRD) data involved a correction for the NERRD effect, as outlined in Figure S13. In brief, the detector parameters were used to back-calculate *R*_1ρ_ rate constants at all RF fields used in the BMRD experiment, and subtracted from the experimental rate constants. These rate constants are close to zero. Analysis of the nonflat profiles (shown in Figure 4) was done with the program relax,^92^ used on the NMRbox server.^93^ As the program cannot handle negative relaxation rate constants, which result from the above-described subtraction procedure, a constant value (5 s^–1^) was added to all data. This plateau value is of no influence for the data analysis, because in the fit procedure the plateau value is fitted and generally not interpreted.

### Preparation of Reduced Tsa1 Samples

In order to obtain a reduced sample, 2.1 mg of [U–^2^H,^15^N,^13^C] Tsa1^WT^ were incubated at 30 °C for 30 min with 5 mM DTT. The protein solution was quickly centrifuged at 10,000*g* in a benchtop centrifuge to pellet down eventual aggregates. Protein supernatant was sedimented into a 1.3 mm rotor overnight by ultracentrifugation at 50,000*g*, 6 °C. The protein sample showed stability in a reduced state for several weeks, as evidenced by 2D hNH MAS NMR experiments.

### Size-Exclusion Chromatography-Multiangle Light Scattering

SEC-MALS experiments were performed using a Superdex Increase 200 10/300 GL column (GE Healthcare) on an Agilent 1260 HPLC Infinity II in phosphate buffer (PBS or 50 mM KPi KCl pH 7.4) at RT (ca. 297 K). Protein elution was monitored by three detectors in series namely, an Agilent multiwavelength absorbance detector (absorbance at 280 and 254 nm), a Wyatt miniDAWN TREOS multiangle light scattering (MALS) detector, and a Wyatt Optilab rEX differential refractive index (dRI) detector. The column was pre-equilibrated overnight in running buffer to obtain stable baseline signals from the detectors before data collection. Molar mass, elution concentration, and mass distributions of the samples were calculated using the ASTRA 7.1.3 software (Wyatt Technology). A BSA solution (2–4 mg/mL), purchased from Sigma-Aldrich and directly used without further purification, was used to calibrate interdetector delay volumes, band broadening corrections, and light-scattering detector normalization using standard protocols within ASTRA 7.1.3.

## Data Availability

The NMR chemical shift assignments of Tsa1 in the decameric oxidized state, decameric reduced state by MAS NMR and of Tsa1^S78D^ by solution-state NMR have been deposited in the BioMagResBank (accession numbers 51788, 51825, 51943). Raw data and fitted parameters (^15^N MAS NMR and solution NMR relaxation data, methyl order parameters of Tsa1^S78D^, relaxation-dispersion data, 2D spectra and analysis scripts/jupyter notebooks) are deposited at the IST Austria Research Explorer data repository and are available at https://www.doi.org/10.15479/AT:ISTA:12820.

## Supporting Information Available

Schematic overview of the reaction cycle of Tsa1; size-exclusion chromatography data of wild-type and S78D mutant Tsa1; 2D solution-NMR ^1^H–^15^N correlation spectra of wild-type and S78D mutant Tsa1; example strips from 3D MAS NMR assignment experiments; secondary structures from NMR assignments (chemical shifts); scanned SDS-PAGE electrophoresis gels; 1D ^1^H MAS NMR spectra of decameric Tsa1^ox^ and Tsa1^red^; MAS NMR ^1^H–^15^N spectra with J-coupling and dipolar-coupling transfer; all MAS NMR ^15^N NERRD and Bloch-McConnell relaxation-dispersion profiles; a figure illustrating the correction of Bloch-McConnell profiles for NERRD effects; solution-NMR Ile, Leu, Val spectra of the dimeric Tsa1^S78D^ mutant with atom-specific assignments; diffusion-ordered solution-NMR spectroscopy (DOSY) data of wild-type and S78D mutant Tsa1; solution-NMR ^15^N relaxation data of Tsa1^S78D^; structural-frustration data for a related peroxiredoxin; analyses of crystallographic structures of related peroxiredoxins. (PDF)
