# Supplementary material for: Disulfide-Bond-Induced Structural Frustration and Dynamic Disorder in a Peroxiredoxin from MAS NMR
Source: J Am Chem Soc. 2023 May 4;145(19):10700–11. doi: 10.1021/jacs.3c01200 (PMC10197130; doi:10.1021/jacs.3c01200)
Supplement: Supplementary file 1 — ja3c01200_si_001.pdf [file ja3c01200_si_001.pdf]

Supporting Information

Disulfide-bond-induced structural frustration and dynamic disorder in a peroxiredoxin from MAS NMR

Laura Troussicot[1,2,4,5], Alicia Vallet[4], Mikael Molin[1,3], Björn M. Burmann[1,2], Paul Schanda[5]

[1]Department of Chemistry and Molecular Biology, University of Gothenburg, SE-405 30 Göteborg, Sweden
[2]Wallenberg Centre for Molecular and Translational Medicine, University of Gothenburg, SE-405 30 Göteborg, Sweden
[3]Department of Life Sciences, Chalmers University of Technology, SE-405 30 Göteborg, Sweden
[4]Institut de Biologie Structurale, Univ. Grenoble Alpes, CEA, CNRS, IBS, 71 Avenue des Martyrs, F-38044 Grenoble, France
[5]Institute of Science and Technology Austria, Am Campus 1, A-3400 Klosterneuburg, Austria

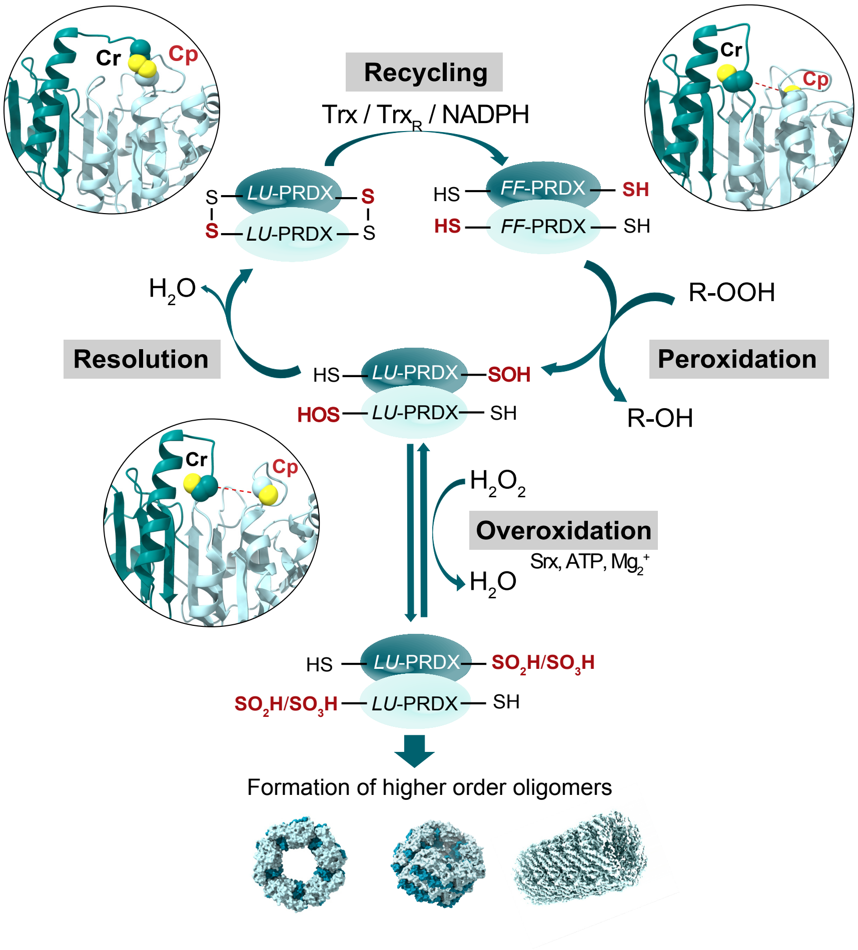

Fig. S1. Schematic representation of the catalytic cycle of peroxiredoxins. See also a recent review on the topic (6).

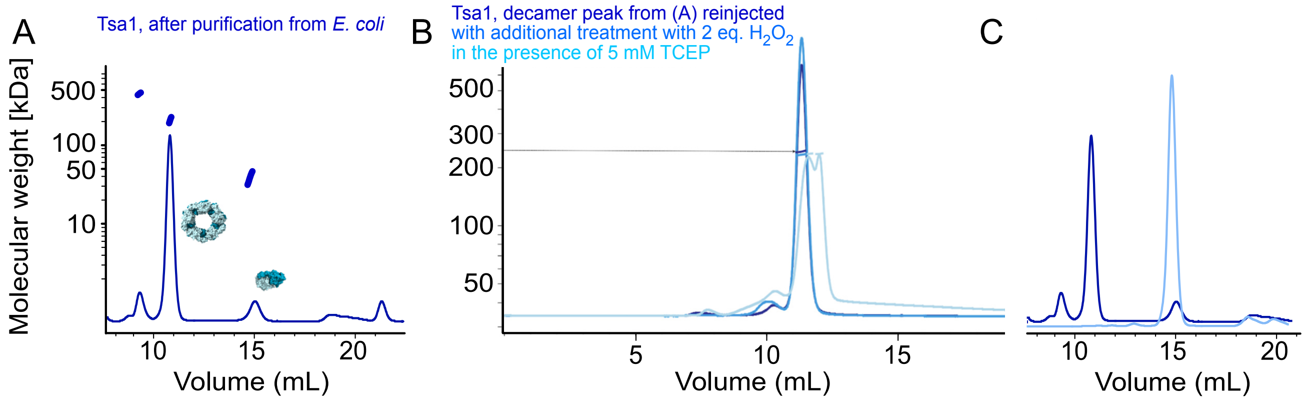

Fig. S2. Size-exclusion chromatography coupled to multi-angle light scattering (SEC-MALS) characterisation of wild-type Tsa1, showing the retention volume (x axis) and the absorbance (continuous lines) and the molecular weight obtained from scattering (short lines at the elution peaks). (A) SEC-MALS profile for Tsa1 obtained at the end of the purification of the protein from *E. coli* (see Methods). This data shows that the protein forms predominantly decameric species (ca. 216 kDa), and a small amount of 20-mer and dimer species. (B) Re-injection of the decamer peak from (A) without treatment (dark blue) and with oxidative or reductive treatment. This data shows that the oligomerisation state of the protein is the same in both oxidized and reduced states. (C) SEC profile of the S78D mutant (light blue). For comparison, the profile of the wild-type protein is shown (dark blue).

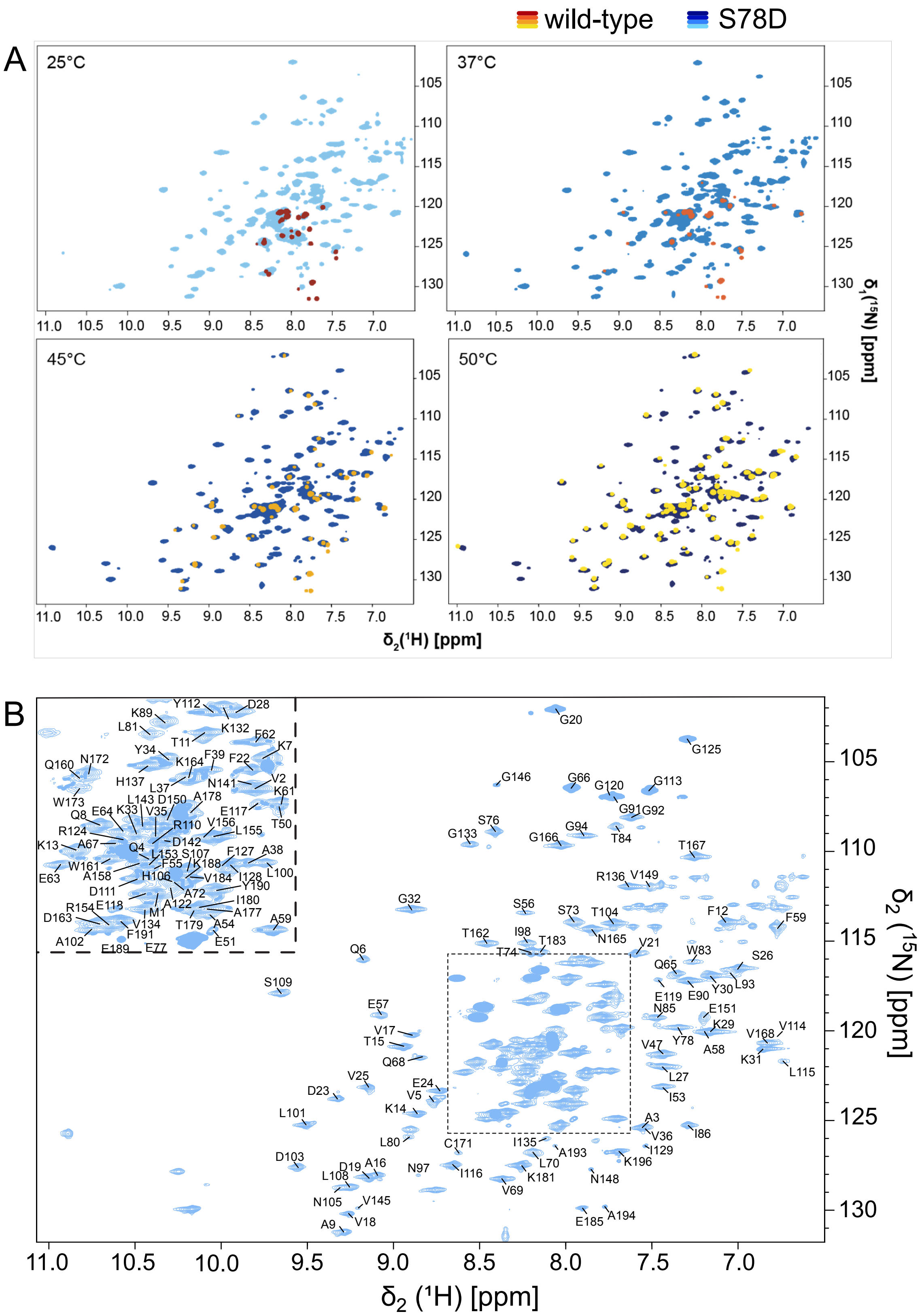

Fig. S3. (A) Solution-state ^{1}H-^{15}N correlation spectra of Tsa1^{WT} and Tsa1^{S78D} recorded at 600MHz at different temperatures, as indicated. (B) Spectrum of Tsa1^{S78D} at 37 °C with sequence-specific resonance assignments.

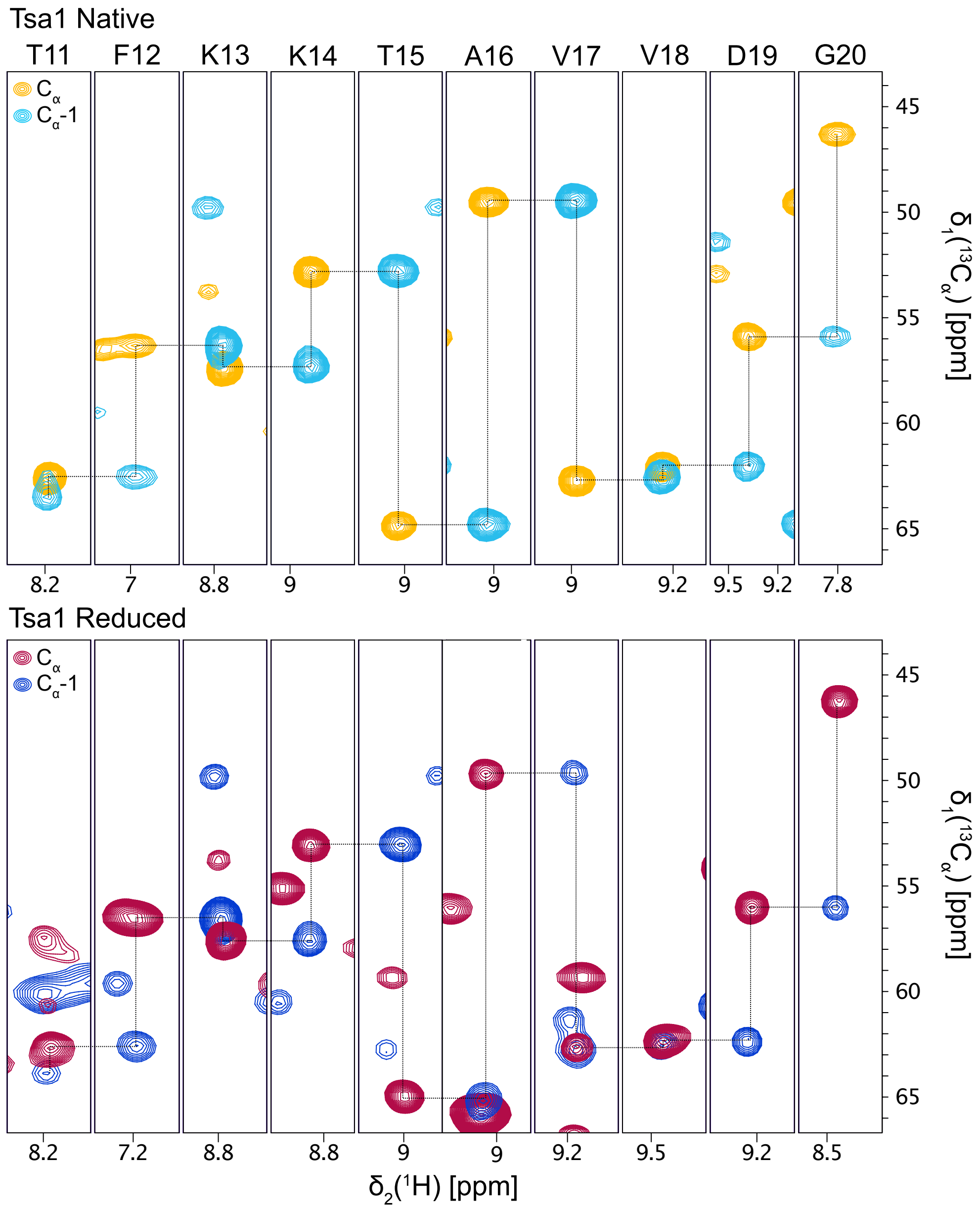

Fig. S4. Representative strips of 3D assignment experiments, resonances for residues T11 to G20 from 3D hCANH and 3D hCAcoNH experiments recorded at 600MHz with 55kHz MAS spinning rate in a 1.3mm rotor, showing sequential assignment of Cαand Cα-1 of Tsa1^{WT} in its native (oxidised) state (top panel) and Tsa1^{WT} in the reduced state (bottom panel).

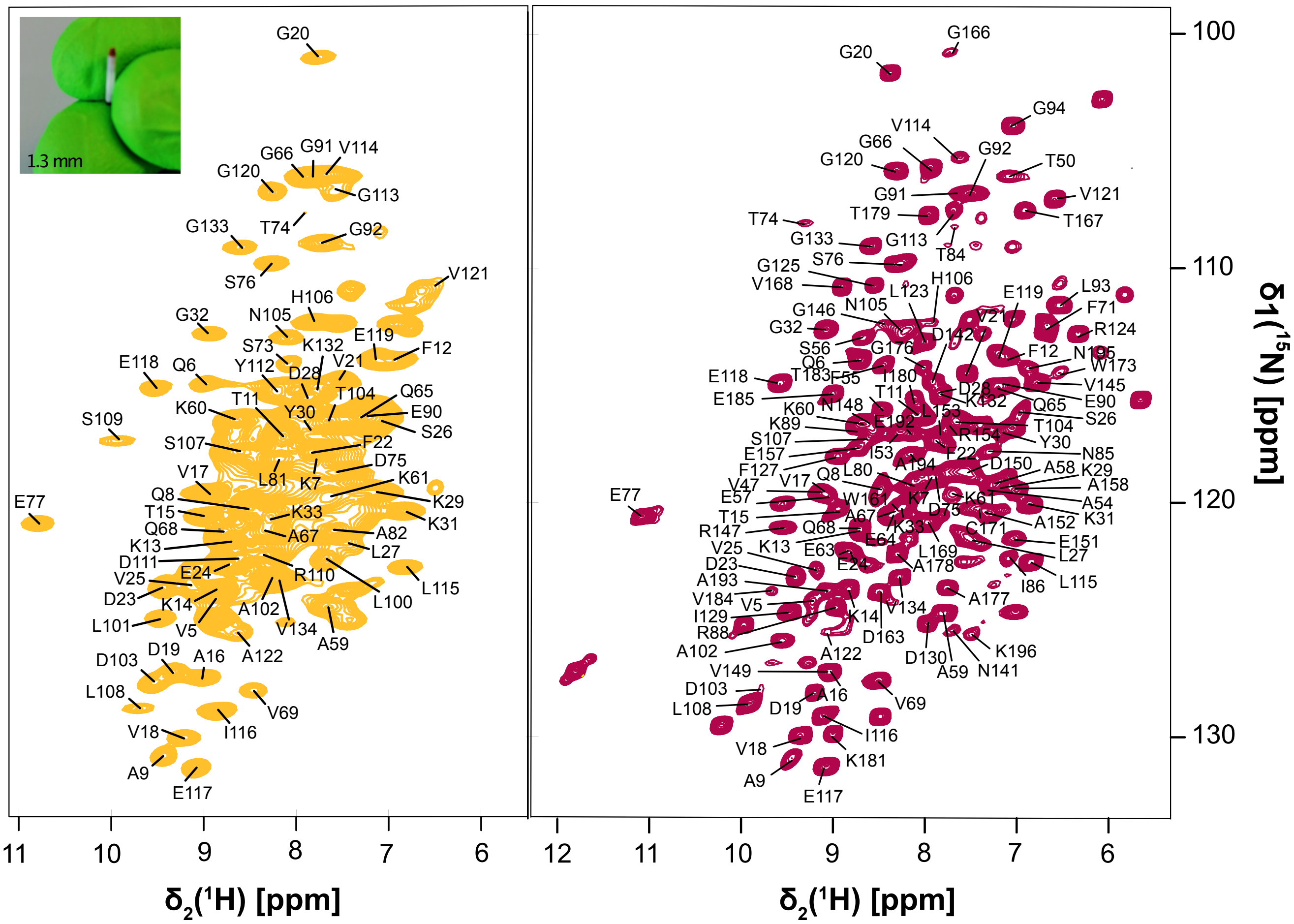

Fig. S5. Magic-angle spinning ^{1}H-^{15}N correlation spectra (based on CP transfer) of Tsa1, with sequence-specific resonance assignments measured at 600MHz, 55kHz MAS in a 1.3 mm rotor. Left: Tsa1^{WT} in native phosphate buffer (without any reducing agent). Right: Tsa1^{WT} in the reduced state in the presence of DTT.

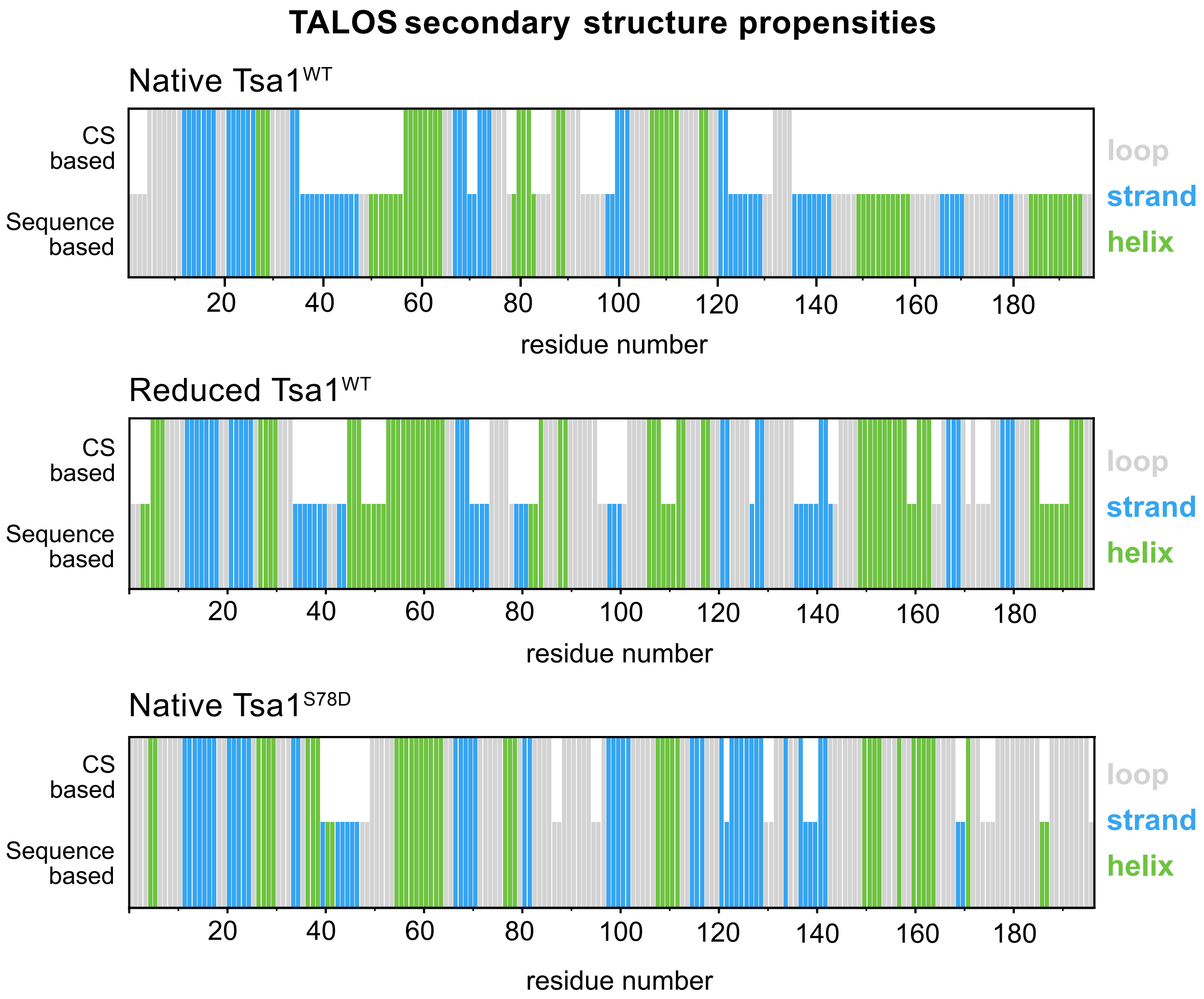

Fig. S6. Secondary structures determined from chemical-shift assignments in decameric Tsa1 in the oxidised state (upper; corresponding to the orange spectrum in Fig. 1A; MAS NMR), decameric Tsa1 in the presence of reducing agent (middle; dark red spectrum of Fig. 1A; MAS NMR) and the dimeric mutant Tsa1^{S78D} in solution state (lower). TALOS reports secondary-structure propensities based on chemical shift of amide-^{1}H, ^{15}N and ^{13}Cα, ^{13}Cβ and ^{13}CO; in the absence of chemical-shift assignments it predicts the secondary structure based on sequence (indicated by shorter bars).

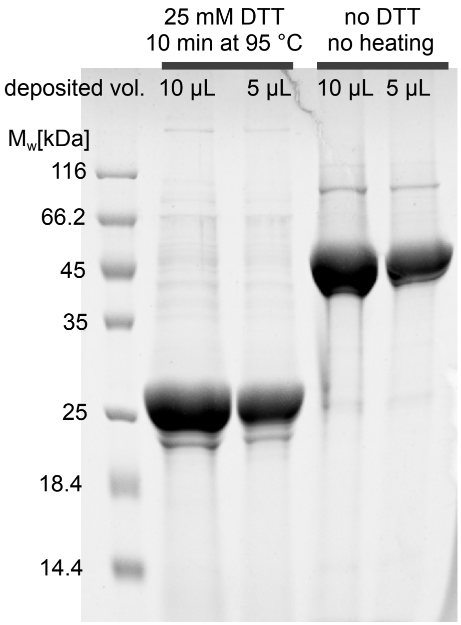

Fig. S7. Tsa1, as obtained after purification from *E. coli* is in a disulfide-bonded state, as evidenced by SDS-PAGE analysis. Shown is the SDS-PAGE of Tsa1 that has been treated with DTT (two left lanes) or which has not been treated (right two lanes). The fact that Tsa1 migrates as a dimer without treatment and as a monomer with DTT treatment shows that the (intermolecular) disulfide bond is present in the sample obtained from purification.

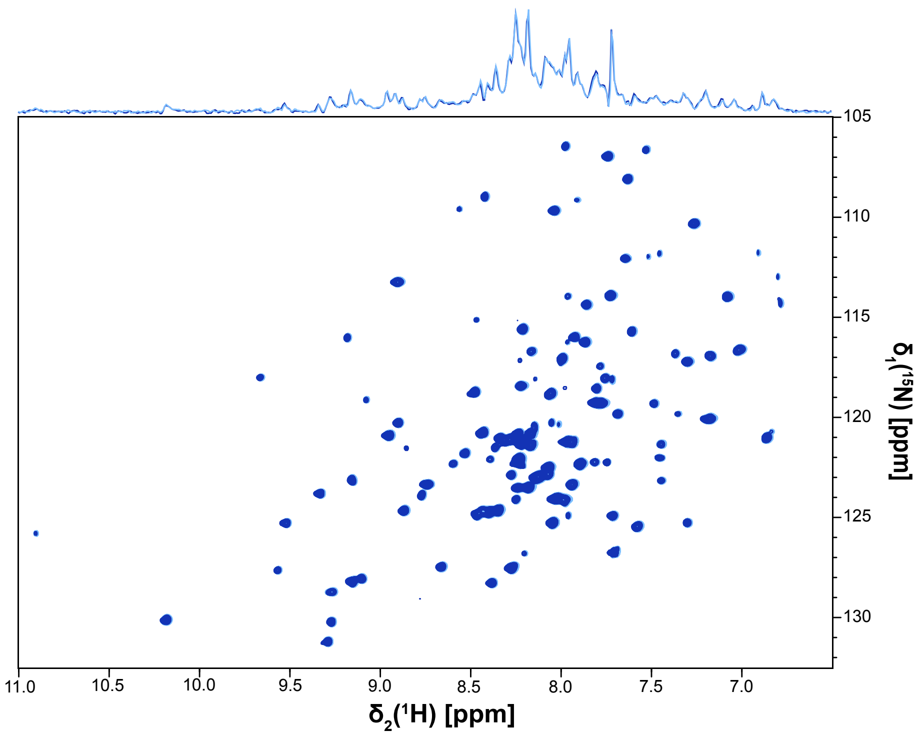

Fig. S8. The native state obtained after expression and purification is an oxidized state. Shown are the 2D ^{1}H-^{15}N spectra of Tsa1^{S78D} recorded at 600MHz and a temperature of 37 °C after purification (light blue), and after incubation with 1 mM of H_2O_2(dark blue), as well as the 1D trace. The protein has a concentration of 500 uM in 50 mM KPi, 50 mM KCl buffer pH 7.4.

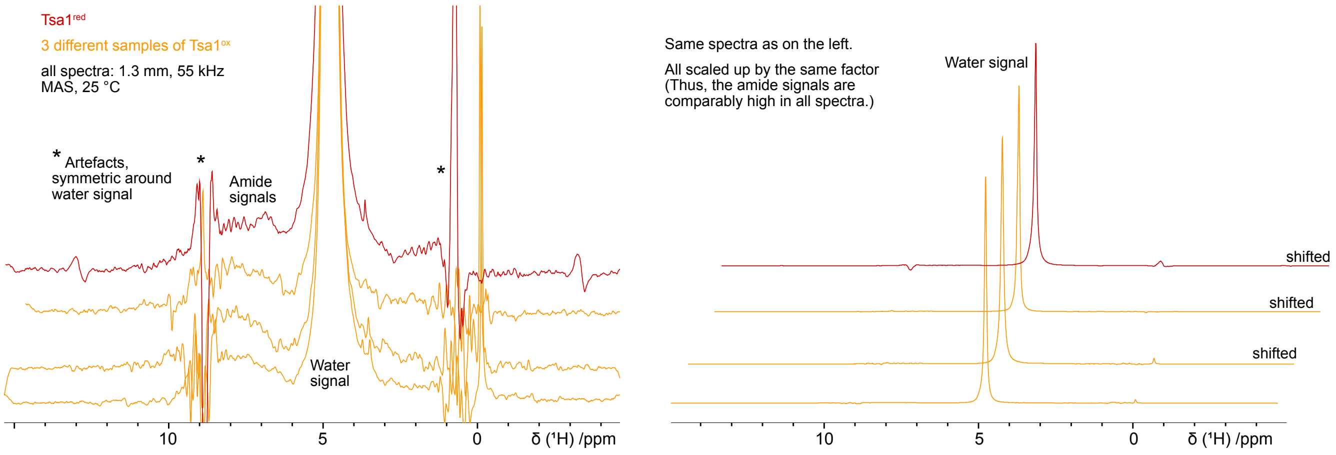

Fig. S9. One-pulse proton-excitation spectra of samples of Tsa1^{ox} (orange) and Tsa1^{red} (red) MAS NMR samples. The same four spectra are shown on the left and right, with different vertical scaling to see either the amide signals (left) or the full water peak (right). All four spectra were scaled by the same factor to go from the representation on the left to the one on the right. These spectra show that the amount of water is comparable in different samples (1.3 mm MAS NMR rotors) of reduced and oxidised Tsa1. The artefacts, denoted by an asterisk in the left spectra, are observed in all our 1.3 mm probes that contain a strong solvent peak; they are symmetric around the water peak, but their exact origin and position is not clear to us.

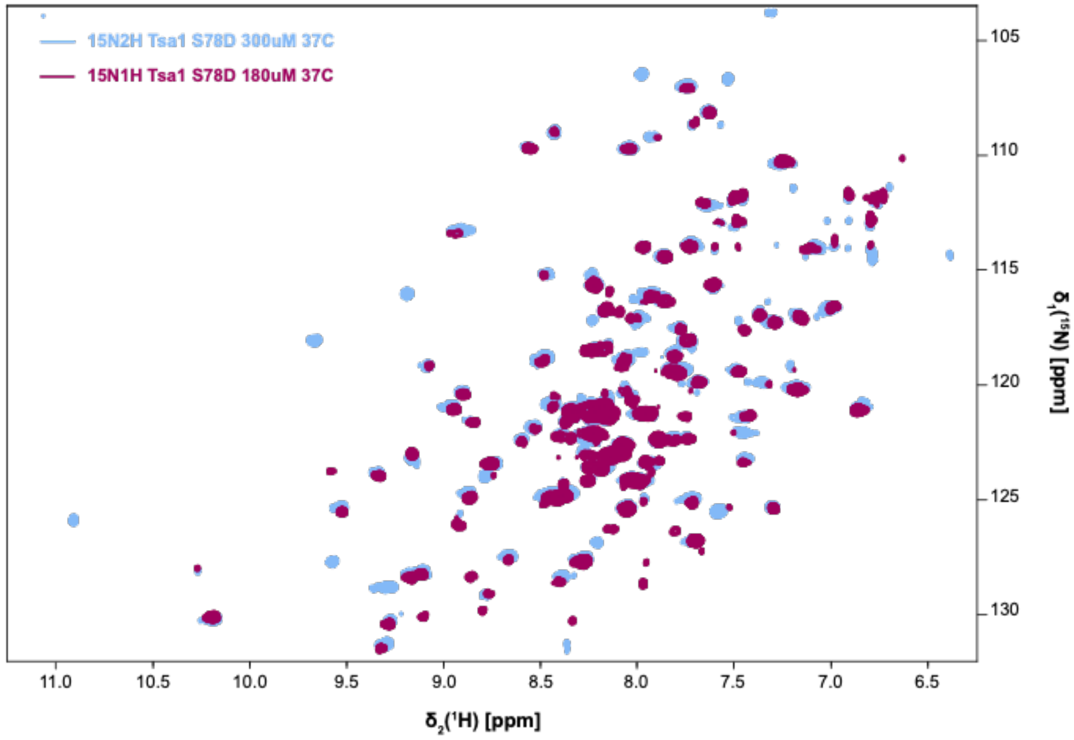

Fig. S10. Comparison of [^{15}N-^{1}H] TROSY HSQC solution-NMR spectra of a deuterated sample, produced in D_2O-based growth medium ([U-^{2}H,^{15}N] Tsa1^{S78D}, light blue), and a protonated sample ([U-^{15}N] Tsa1^{S78D}, purple) recorded at 600MHz and 37 °C in phosphate buffer. As the [U-^{2}H,^{15}N]-labelled sample was produced in a bacterial culture in D_2O, amides which are not solvent accessible (i.e., they do not get re-protonated) would be invisible in this ^{15}N-^{1}H spectrum. Thus, if amide re-protonation was incomplete, one would expect peak in the spectrum of the [U-^{15}N] sample which are absent in the [U-^{2}H,^{15}N]. This is not the case, which indicates that re-protonation is achieved during the purification of the protein in H_2O.

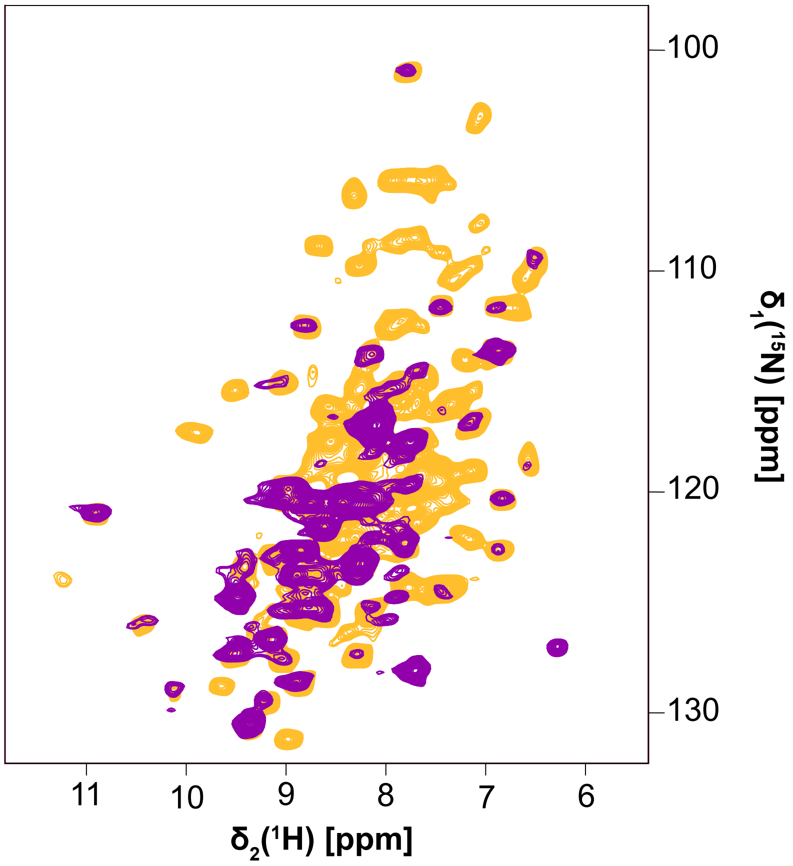

Fig. S11. Overlay of 2D ^1H-^{15}N spectra of sedimented [U-^2H,^{13}C,^{15}N] Tsa1^{WT} in 50 mM potassium phosphate, 50 mM KCl, pH 7.4, recorded with a 50 kHz MAS frequency using a CP transfer (orange) or an INEPT transfer (purple). If large-scale ps-ns motions were present, transfer with the CP scheme would be strongly reduced while INEPT transfer would be expected to be efficient; hence, peaks missing in the CP experiments may appear in the INEPT experiment. It is evident that the large number of peaks missing in the CP-based spectrum does not appear in the INEPT spectrum, suggesting that the peaks missing in the spectra of Tsa1^{ox} are missing because of µs dynamics, which broadens resonances beyond detection. This is fully in agreement with the quantitative measurements of dynamics, in particular the NERRD data. Note that there are two peaks in the INEPT spectrum which are not found in the CP spectrum, which likely are very flexible sites. We do not have assignments for these two peaks.

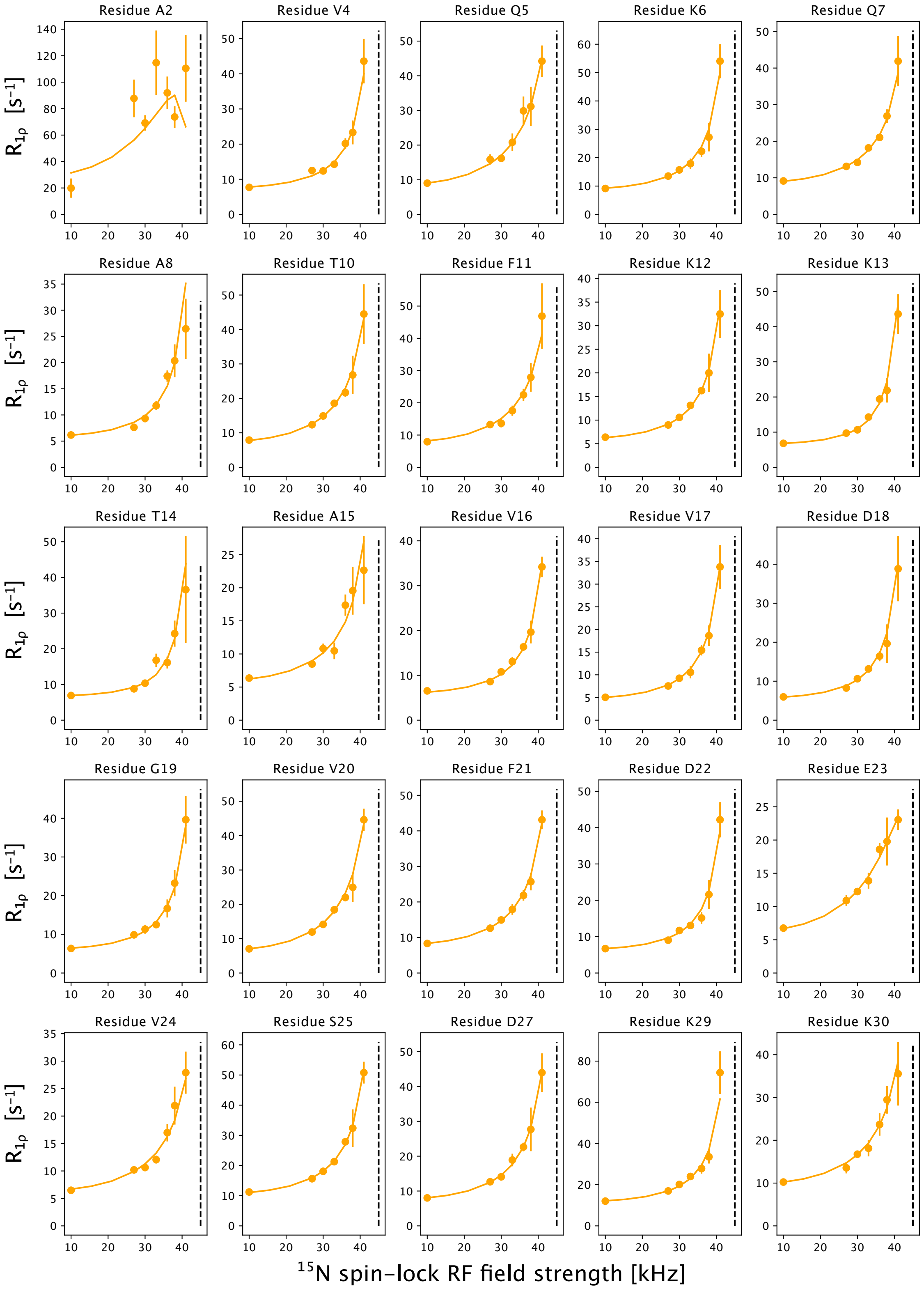

Fig. S12. ^{15}N $R_{1\rho}$ NEar-Rotary-resonance Relaxation Dispersion data of oxidised Tsa1 in the solid state. The solid line indicates the back-calculated $R_{1\rho}$ rate constants using the detectors fit with four components (see Fig. 3). The dashed vertical line indicates the n=1 rotary-resonance condition, i.e., where the RF field strength equals the MAS frequency.

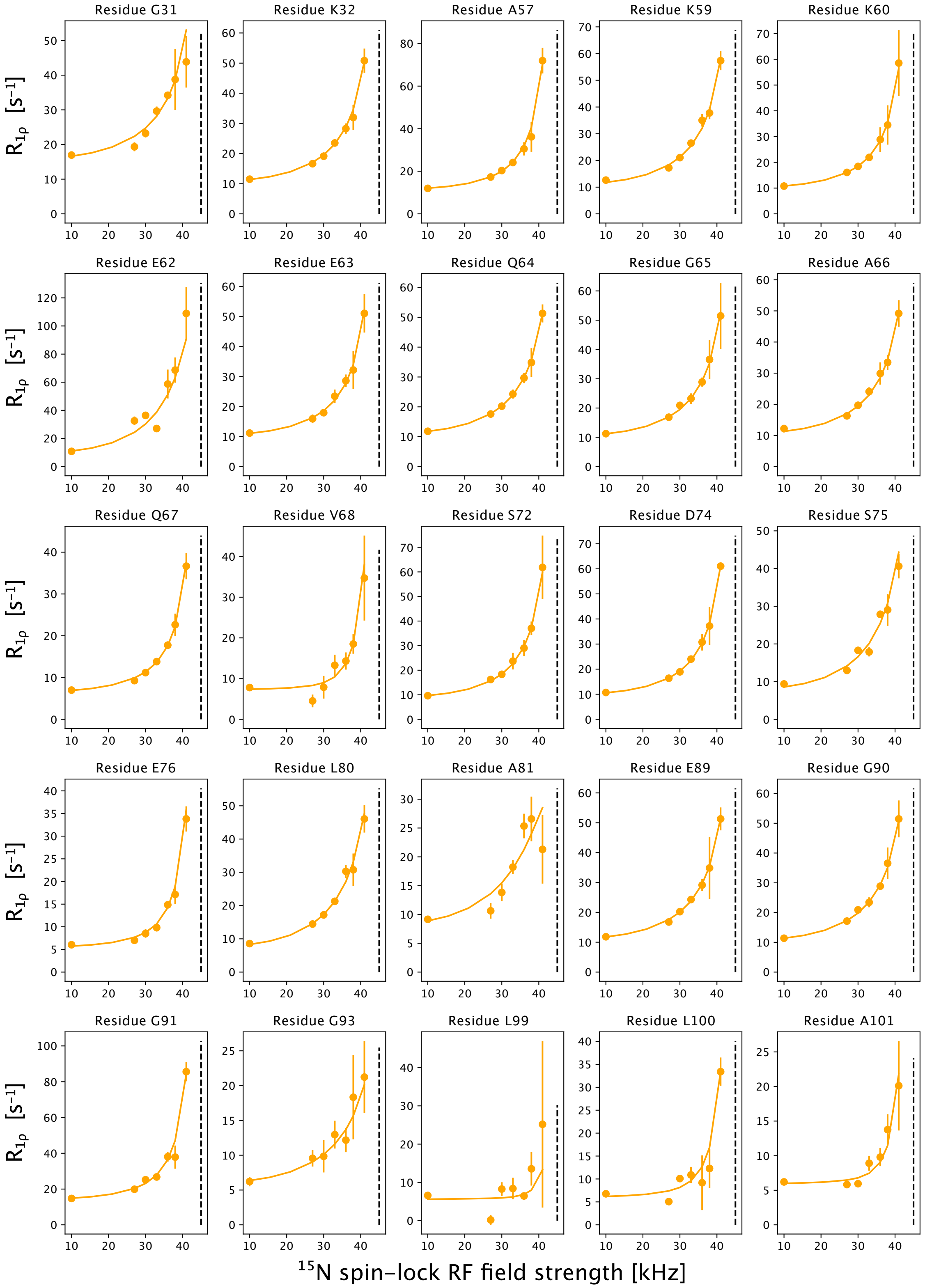

Fig. S12. (continued) ^{15}N $R_{1\rho}$ NEar-Rotary-resonance Relaxation Dispersion data of oxidised Tsa1 in the solid state.

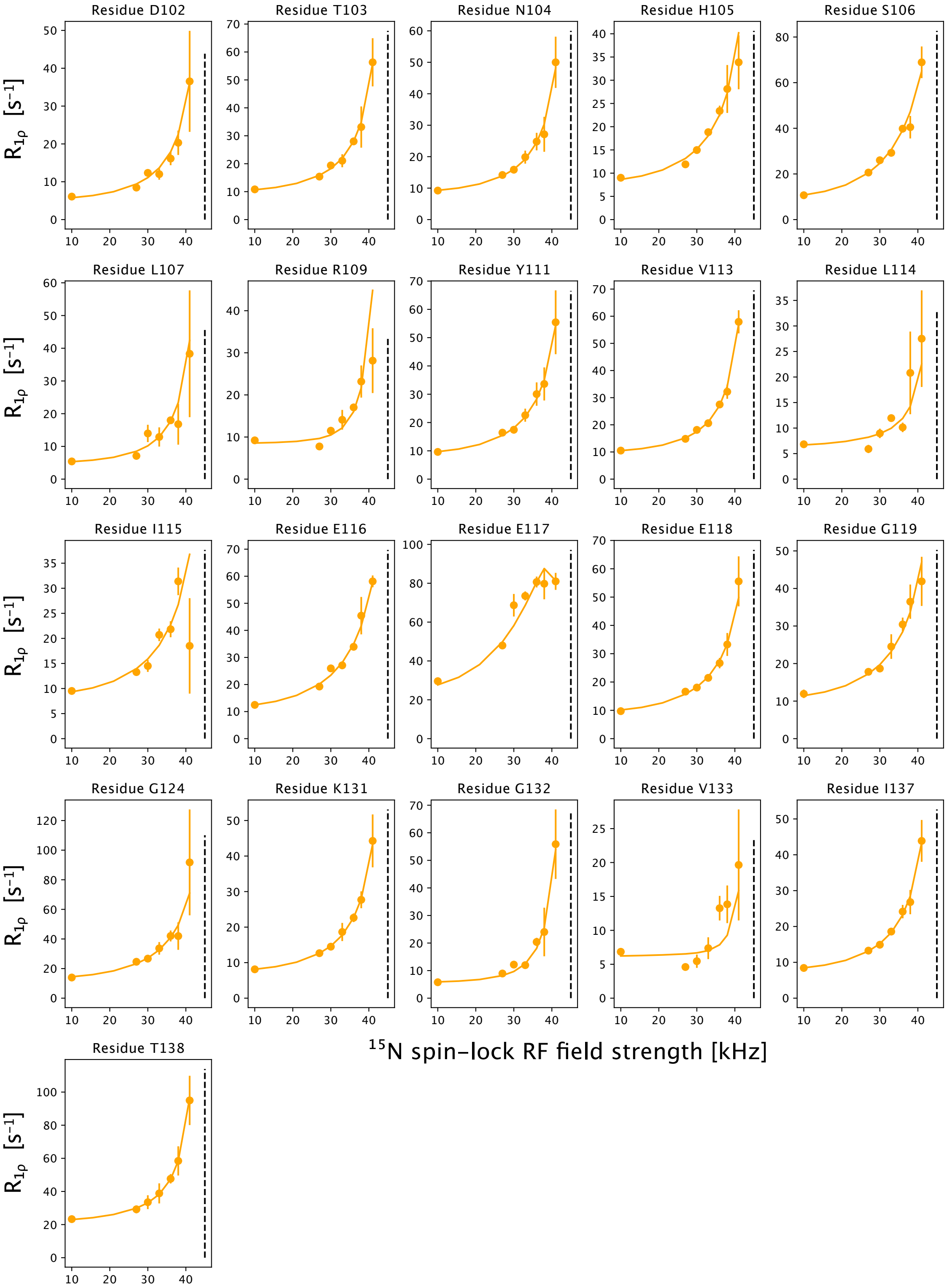

Fig. S12. (continued) ^{15}N $R_{1\rho}$ NEar-Rotary-resonance Relaxation Dispersion data of oxidised Tsa1 in the solid state.

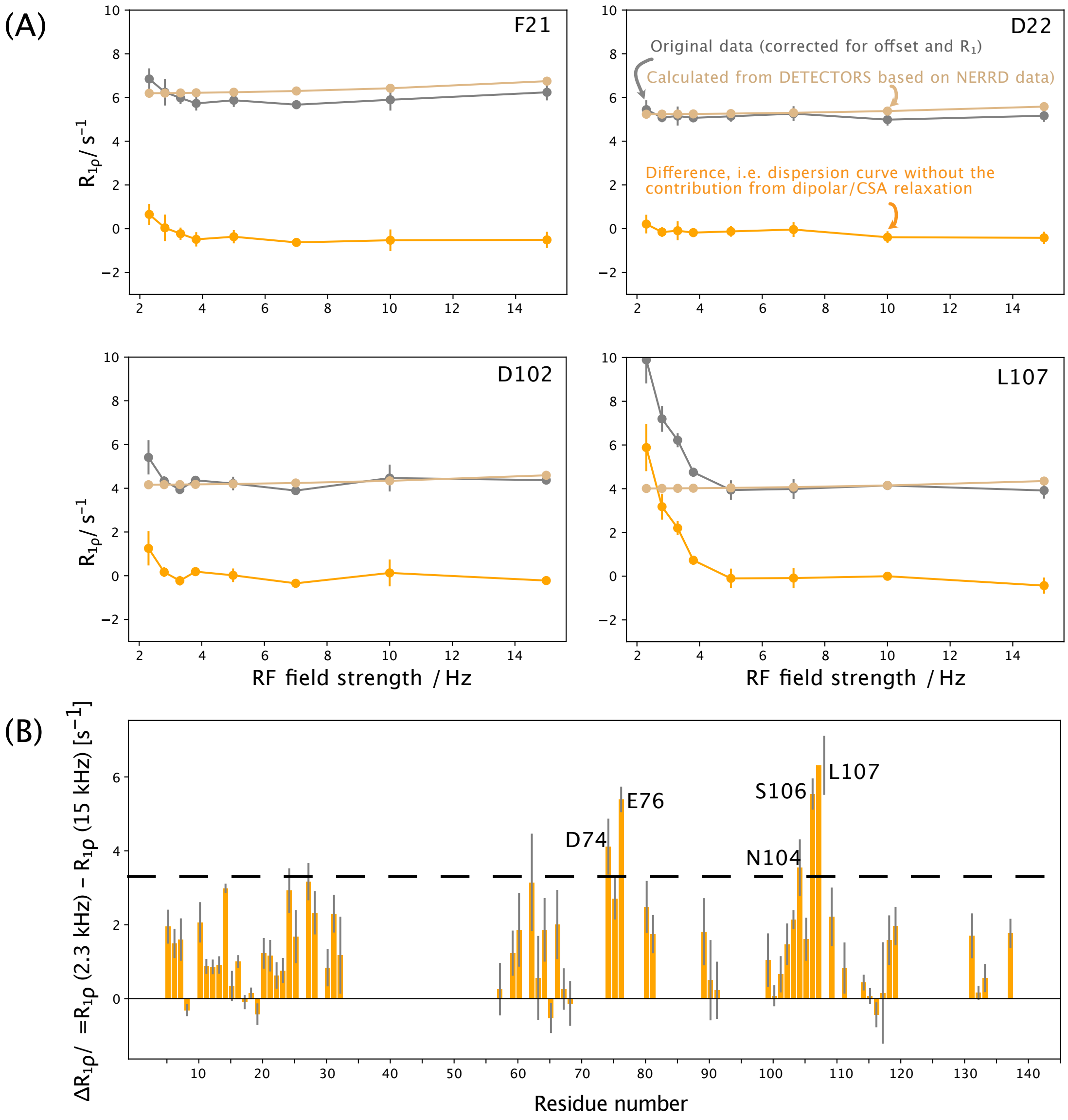

Fig. S13. Analysis strategy and data selection of Bloch-McConnell $R_{1\rho}$ relaxation dispersion data. (A) Outline of the procedure by which the dipolar and CSA relaxation has been accounted for in the Bloch-McConnell relaxation-dispersion profiles. The dipolar and CSA relaxation (the "tail" of the NERRD profile) contributes still to the low-RF field $R_{1\rho}$ rate constants, which makes analysis of the chemical-shift induced relaxation dispersion complicated, and not possible with the Bloch-McConnell formalism. To account for the dipolar and CSA relaxation, we used the detectors analysis with four components (see solid lines in Fig. 3) and used these fitted parameters to calculate the $R_{1\rho}$ rate constant under the conditions at which the Bloch-McConnell RD data have been measured (55 kHz MAS, RF fields from 2.3 to 15 kHz). We subtracted these back-calculated $R_{1\rho}$ rate constants from the experimentally measured ones. Four examples are shown. The grey data points are the original data; the brown data points are the back-calculated ones and the resulting difference is shown in orange here and also in Fig. S14. The corrected values are close to zero, showing that the detectors analysis, based on $R_{1\rho}$ rate constants measured at 45 kHz MAS and RF fields from 10 to 41 kHz, is in good agreement with these data, which had not been used in the detectors analysis. Because the program used for fitting the BMRD data (relax) cannot handle negative values, we have added a uniform positive offset to these orange data, before fitting the BMRD data.
(B) Difference of $R_{1\rho}$ rate constants at the highest (15 kHz) and lowest RF field (2.3 kHz) of the data set, highlighting the residues with the most significant Bloch-McConnell relaxation-dispersion effect. In the BMRD fits, the five residues labelled in this plot have been used.

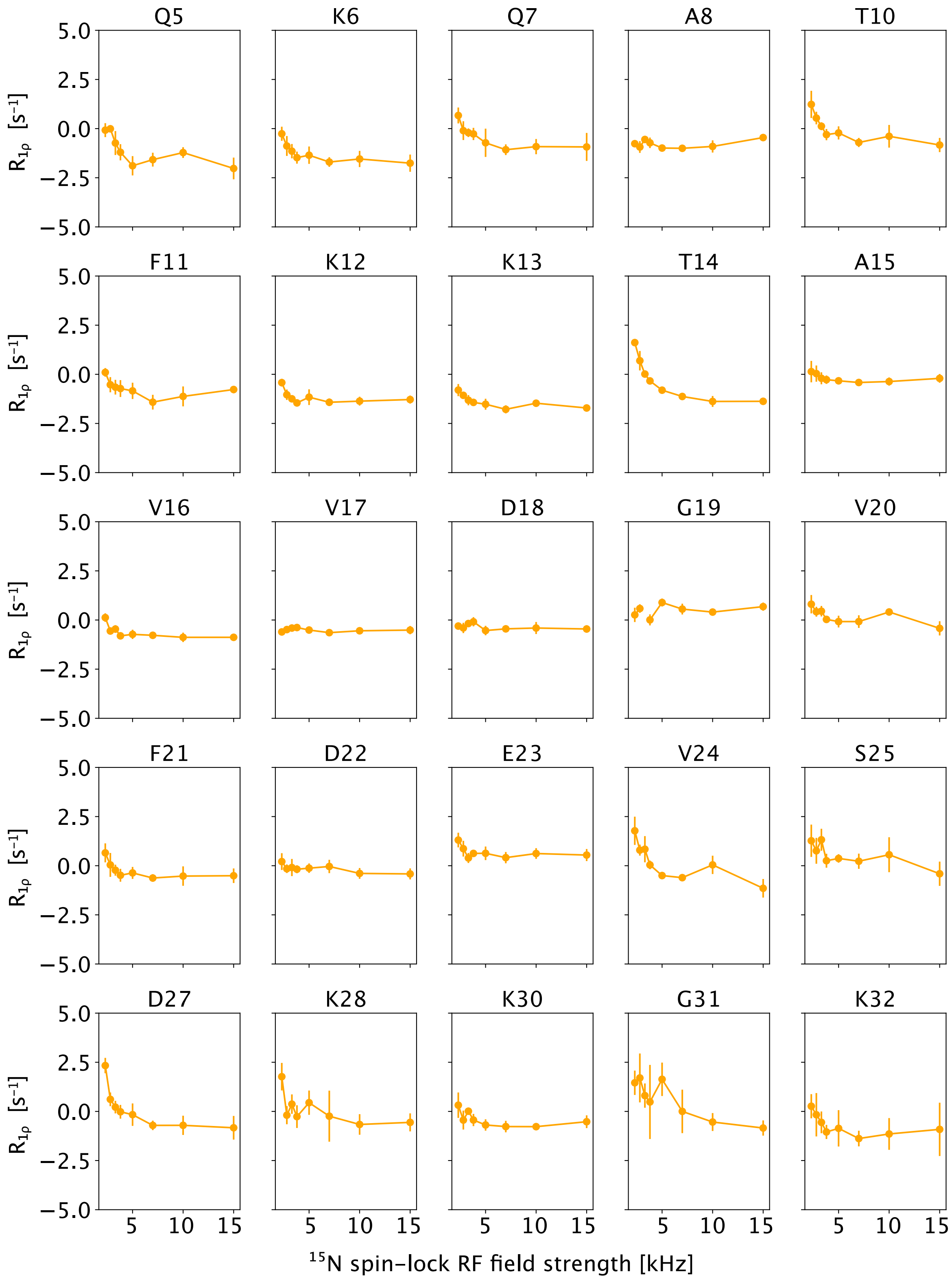

Fig. S14. Bloch-McConnell ^{15}N $R_{1\rho}$ relaxation dispersion curves of Tsa1^{ox}, corrected for resonance offset and R_1; additionally the back-calculated rate constants based on the 4-detectors analysis have been subtracted from the data. See Fig. S13A for examples of this procedure.

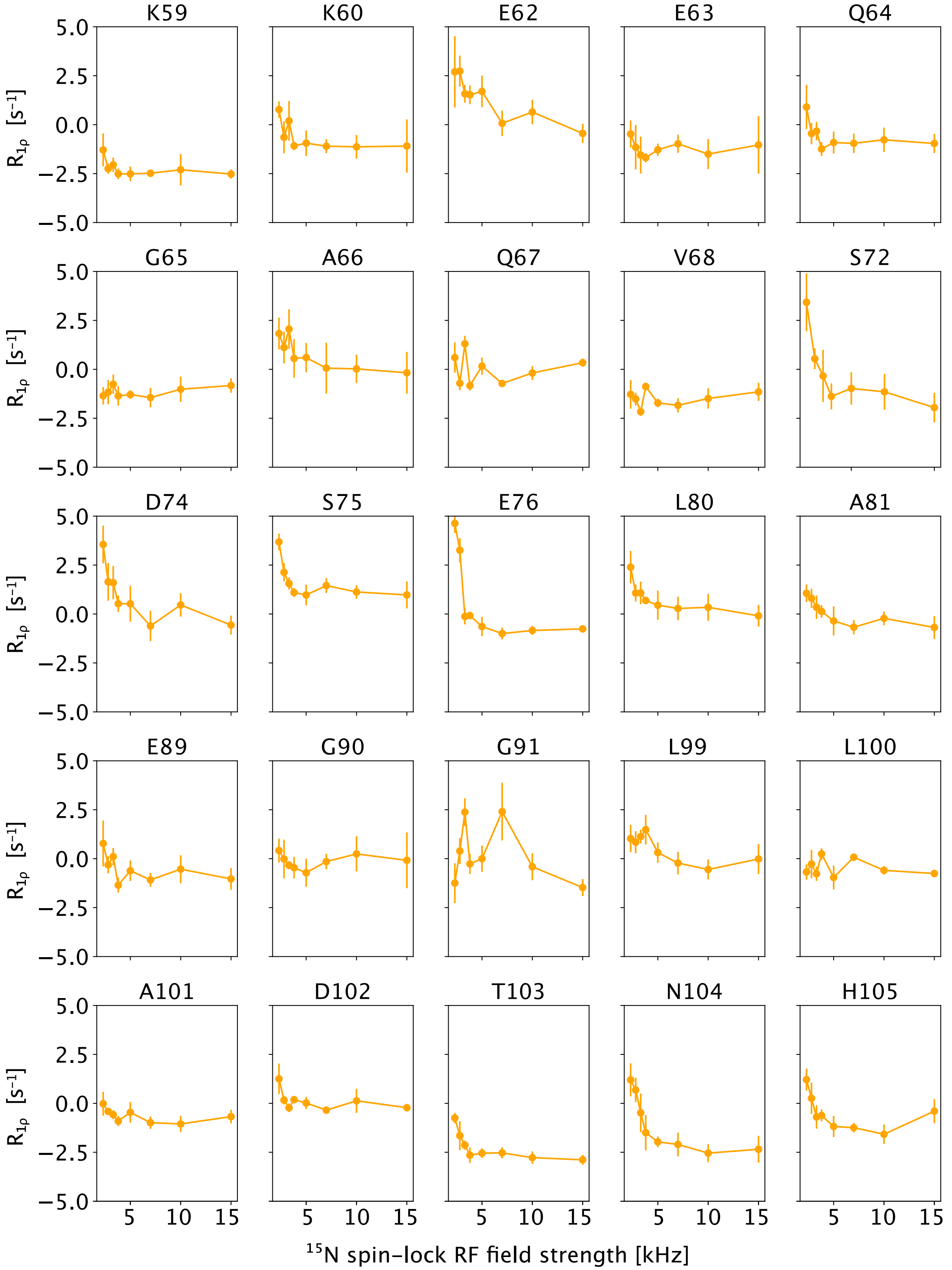

Fig. S14. (continued) Bloch-McConnell ^{15}N $R_{1\rho}$ relaxation dispersion curves of Tsa1^{ox}

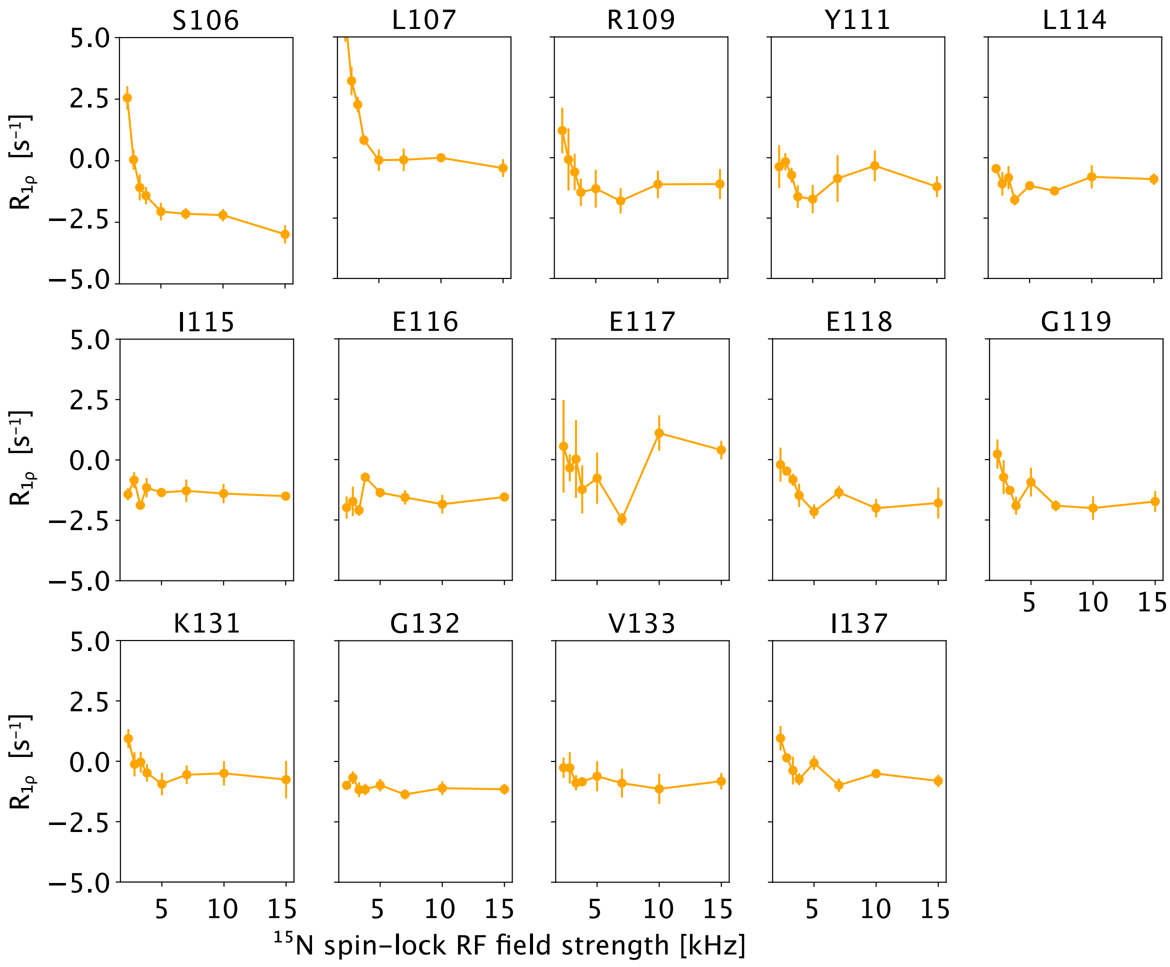

Fig. S14. (continued) Bloch-McConnell ^{15}N $R_{1\rho}$ relaxation dispersion curves of Tsa1^{ox}.

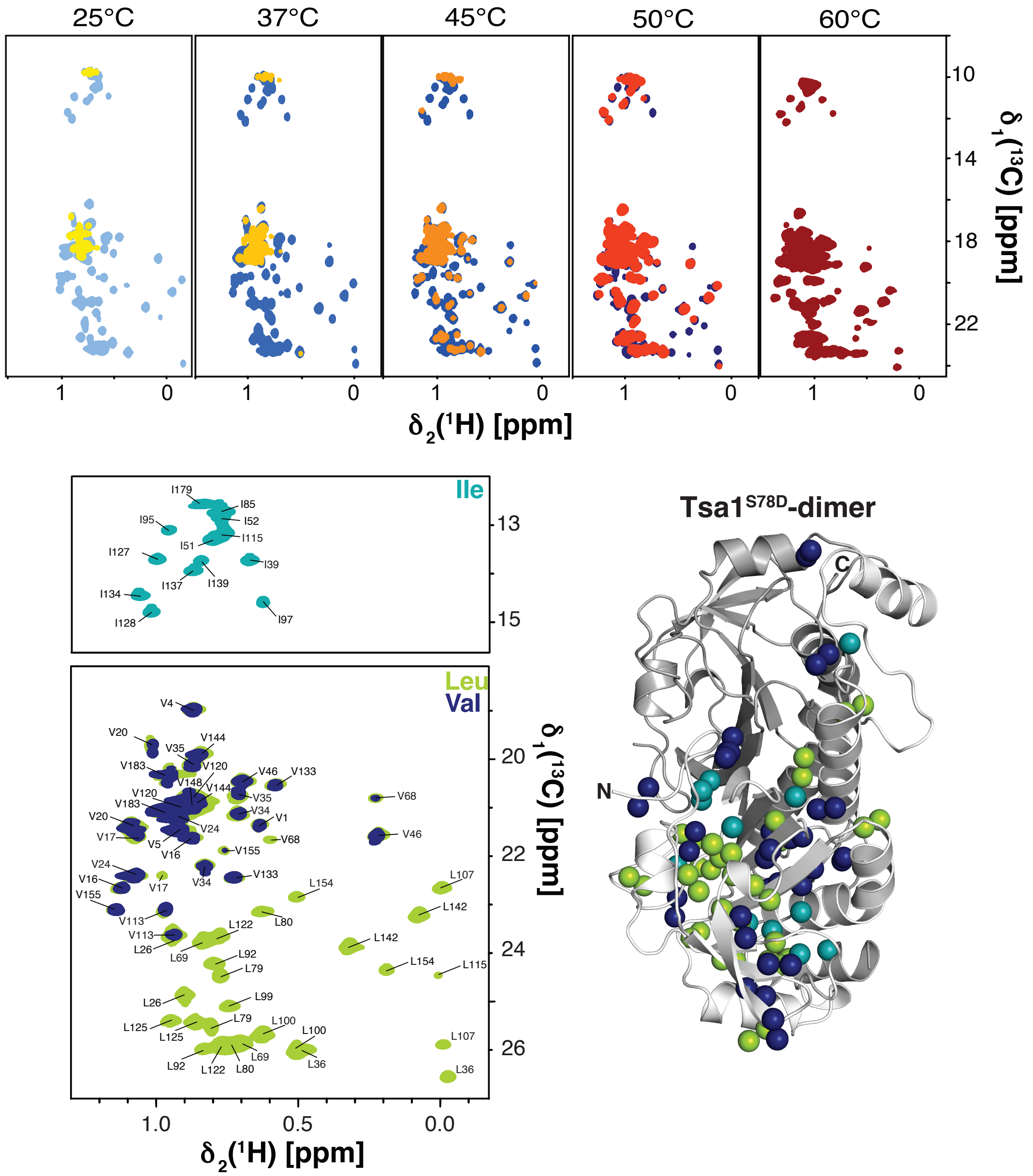

Fig. S15. NMR of methyl labeled Tsa1. (Top) Temperature titration of [U-^{2}H,^{15}N, Ile-δ1-^{13}CH$_3$, Leu/ Val-^{13}CH$_3$] labeled Tsa1^{WT} (yellow to red) and [U-^{2}H,^{15}N, Ile-δ1-^{13}CH$_3$, Leu/ Val-^{13}CH$_3$] labeled Tsa1^{S78D} (light blue to dark blue) by solution state NMR, ranging from 25°C to 60°C. Data show that Tsa1^{S78D} is not impacted by temperature change. Therefore its structure is conserved. In contrast, for Tsa1^{WT}, the spectrum only shows signals of few residues at 25°C. These peaks tend to correspond to the most flexible parts of the protein, such as I179, I85 or V183, for which the order parameters are low (see Fig. 5); the large-amplitude motion of these sites increases their coherence life times and it is, thus, expected that they are visible even at low temperature. When increasing the temperature one retrieves the same NMR spectrum as the one obtained for the dimeric mutant. This is understandable as high temperature promotes the dissociation to dimers (see SEC-MALS data in Fig. S2).
(Bottom) 2D ^{1}H-^{13}C spectrum of [U-^{2}H,^{15}N, Ile-δ1-^{13}CH$_3$, Leu/ Val-^{13}CH$_3$] Tsa1^{S78D}, with manual assignment achieved using ^{13}C -methyl-SOFAST-NOESY experiments with mixing times of 50 ms and 600 ms as well as a 3D HMBC-HMQC experiment. Spectra from two different samples are shown in the lower panel, from either labeling Val and Leu simultaneously (green) or from labeling only Val (blue). The right panel shows the location of Ile, Leu and Val methyl groups on a monomeric subunit of the Tsa1 structure.

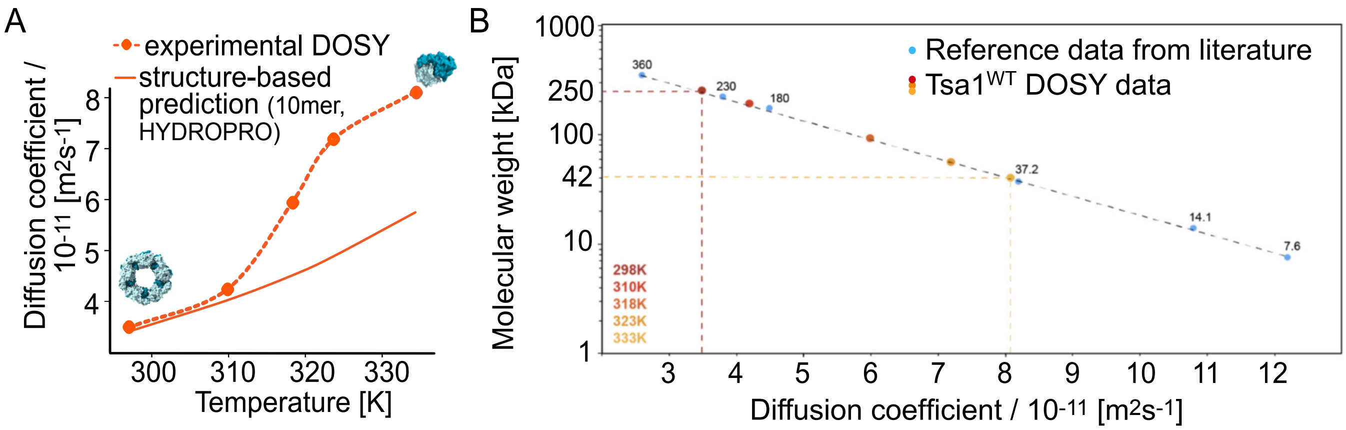

Fig. S16. Diffusion-ordered spectroscopy (DOSY) data measured by methyl-directed experiments of wild-type Tsa1. Left: fitted diffusion coefficients as a function of temperature. The solid orange line is the predicted diffusion coefficient using the program HYDROPRO (78). Right: estimated molecular weights. To construct the calibration curve on the right, the following published translational diffusion coefficients, measured at 25℃, were used: immunoglobin-binding domain of streptococcal protein G, GB1 (MW = 6.2 kDa) (77), lysozyme (MW = 14.1 kDa) and interleukin-10 (MW = 37.2 kDa) (76), bacterial HslV (MW = 230 kDa), one-half proteasome from *Thermoplasma acidophilum*, α7α7 (MW = 360 kDa), and the α7 single ring variant of the proteasome (MW = 180 kDa) (75).

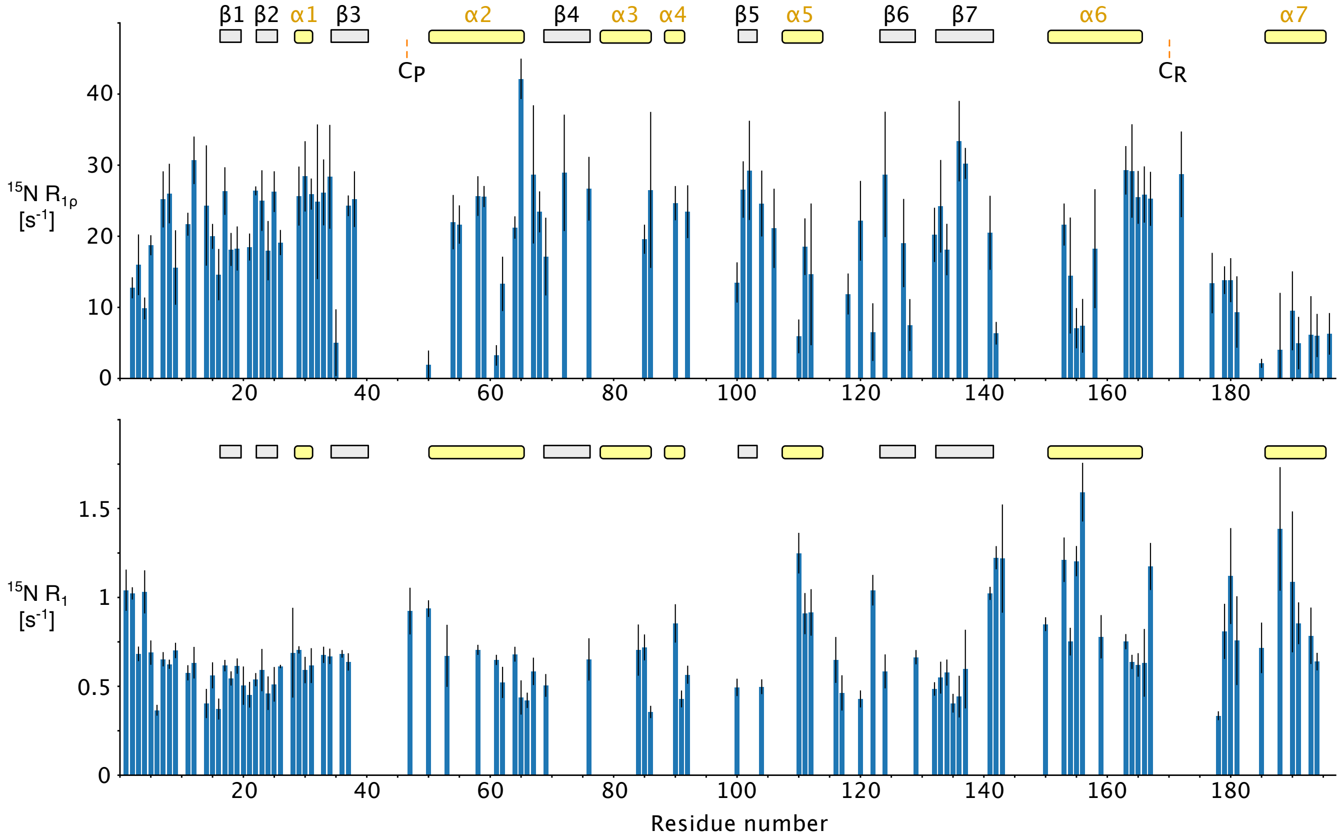

Fig. S17. ^{15}N spin relaxation data of the dimeric Tsa1^{S78D} mutant at 37 °C. The indicated secondary structure elements above the plots are obtained from the decameric state (PDB 3SBC). It is evident that the helix α7 has higher flexibility than the other secondary structure elements. Together with the TALOS secondary-structure data (Fig. 1), this observations shows that in the dimer the C-terminal helix is disordered.

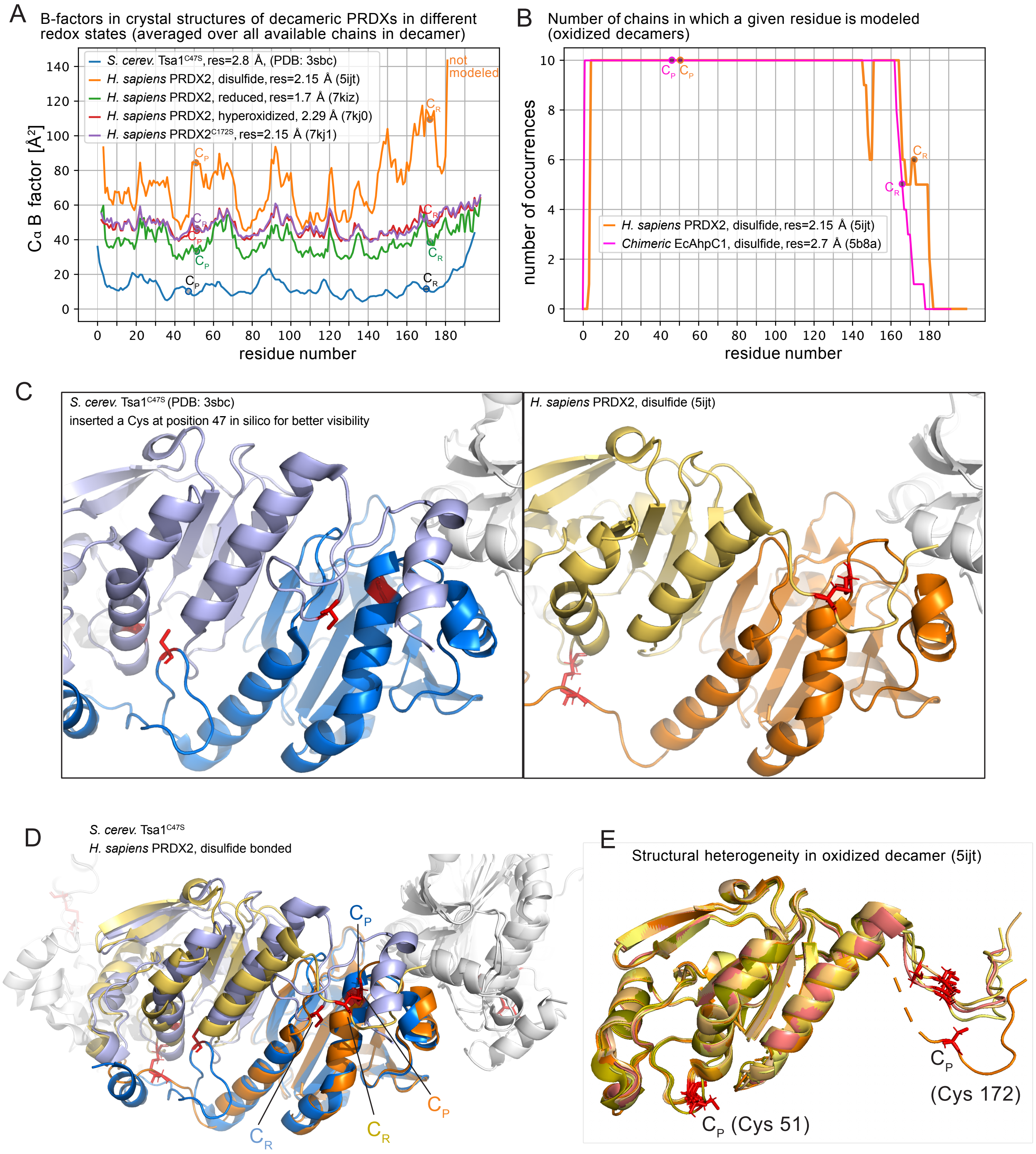

Fig. S18. Insights into PRDXs structure from crystallography. A) B-factors of decameric PRDXs Cαin different oxidation states. B) Occurences of modelized residues over the sequence of the oxidized decamer of hPRDX2, showing that the C-terminal part of the protein cannot be properly modeled because of it intrinsic dynamics or lack of structure. C) Comparison of the α2 helix structure between reduced *Sc* Tsa1 (3SBC) with insertion of the wild type Cys47 for better visibility, and oxidized *Hs* PRDX2 (5IJT) in its S-S state. D) Overlay of figures C for better understanding. E) Structural ensembles of oxidized decamer of *Hs* PRDX2 (5IJT) showing structural heterogeneity of the C-terminal tail.

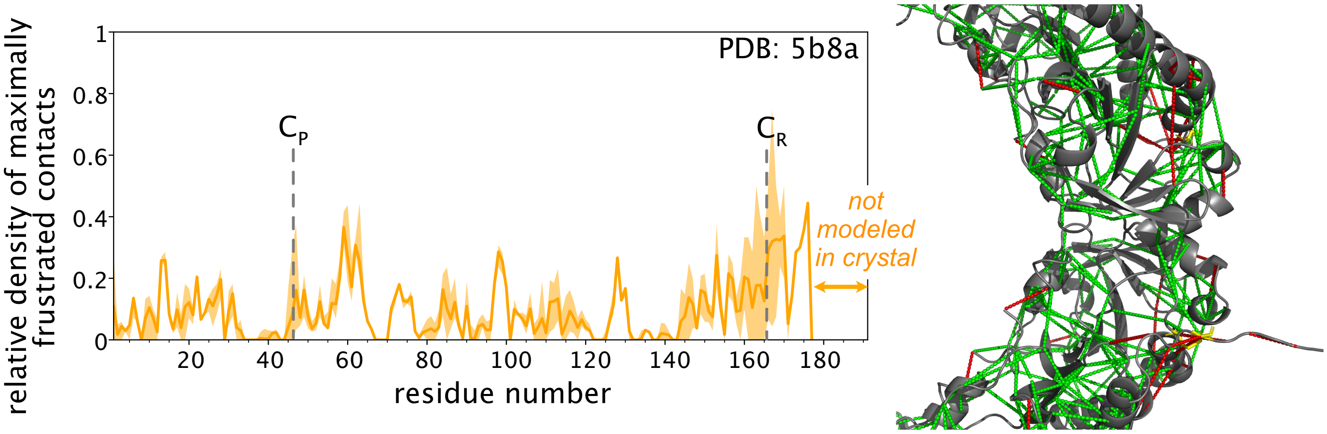

Fig. S19. Structural frustration data of the oxidised PRDX from *E. coli*, PDB entry 5B8A.
